# COZOID: contact zone identifier for visual analysis of protein-protein interactions

**DOI:** 10.1186/s12859-018-2113-6

**Published:** 2018-04-06

**Authors:** Katarína Furmanová, Jan Byška, Eduard M. Gröller, Ivan Viola, Jan J. Paleček, Barbora Kozlíková

**Affiliations:** 1 Faculty of Informatics, Masaryk University, Brno, Czech Republic; 2 Department of Informatics, University of Bergen, Bergen, Norway; 3 Institute of Visual Computing & Human-Centered Technology, TU Wien, Wien, Austria; 4 National Centre for Biomolecular Research, Masaryk University, Brno, Czech Republic; 5 Central European Institute of Technology, Masaryk University, Brno, Czech Republic

**Keywords:** Protein-protein interaction, Contact zone, Visualization

## Abstract

**Background:**

Studying the patterns of protein-protein interactions (PPIs) is fundamental for understanding the structure and function of protein complexes. The exploration of the vast space of possible mutual configurations of interacting proteins and their contact zones is very time consuming and requires the proteomic expert knowledge.

**Results:**

In this paper, we propose a novel tool containing a set of visual abstraction techniques for the guided exploration of PPI configuration space. It helps proteomic experts to select the most relevant configurations and explore their contact zones at different levels of detail. The system integrates a set of methods that follow and support the workflow of proteomics experts. The first visual abstraction method, the Matrix view, is based on customized interactive heat maps and provides the users with an overview of all possible residue-residue contacts in all PPI configurations and their interactive filtering. In this step, the user can traverse all input PPI configurations and obtain an overview of their interacting amino acids. Then, the models containing a particular pair of interacting amino acids can be selectively picked and traversed. Detailed information on the individual amino acids in the contact zones and their properties is presented in the Contact-Zone list-view. The list-view provides a comparative tool to rank the best models based on the similarity of their contacts to the template-structure contacts. All these techniques are interactively linked with other proposed methods, the Exploded view and the Open-Book view, which represent individual configurations in three-dimensional space. These representations solve the high overlap problem associated with many configurations. Using these views, the structural alignment of the best models can also be visually confirmed.

**Conclusions:**

We developed a system for the exploration of large sets of protein-protein complexes in a fast and intuitive way. The usefulness of our system has been tested and verified on several docking structures covering the three major types of PPIs, including coiled-coil, pocket-string, and surface-surface interactions. Our case studies prove that our tool helps to analyse and filter protein-protein complexes in a fraction of the time compared to using previously available techniques.

**Electronic supplementary material:**

The online version of this article (10.1186/s12859-018-2113-6) contains supplementary material, which is available to authorized users.

## Background

Understanding the constitution and biological function of proteins is essential in many research disciplines, such as medicine and pharmaceutics. Most of the proteins critical for cellular life act in a cooperative manner, forming multiprotein complexes. It is estimated that approximately 800 complexes exist in just one yeast cell [1].

All complexes are composed of subunits, which constitute the complex via mutual protein-protein interactions (PPIs). The main goal of studying these PPIs, known as protein-protein docking, is to identify the appropriate spatial configuration of the interacting proteins. This configuration is represented by the mutual spatial orientation of the interacting proteins. Each configuration contains a contact zone, consisting of the set of amino acids from both interacting proteins that are with interaction distance, usually spanning from 3 to 5 Ångströms.

The structure determination of PPIs in laboratories is very challenging, as well as expensive and time-consuming. This is due to many problems related to the dynamic nature of proteins, difficulties in their purification and sample preparation. Therefore, computational docking is often used to study the feasibility of proposed configurations. Many algorithms and tools have appeared to examine these configurations in the last years. A categorization of the existing algorithms, along with a description of their basic principles, was published recently by Huang [2].

However, these algorithms produce a large number of possible configurations, which need to be explored to identify the proteomically most relevant ones. Even though the computational tools usually provide the users with some score to rank the configurations, the resulting ordering does not necessarily correspond to their proteomic relevance. Therefore, the configurations have to be processed and examined manually, which requires a proper visual support to enhance the exploration process.

**Fig. 1:**
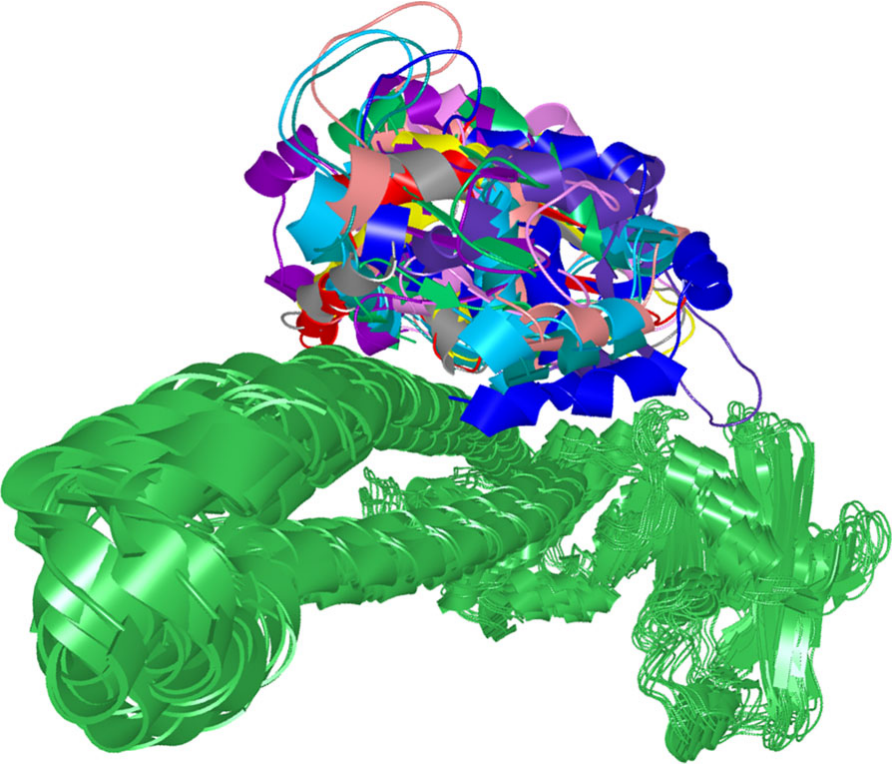
Traditionally used 3D visual representation of configurations. Typical visual representation of configurations used by the proteomic experts that suffers from substantial visual clutter. It superposes several possible configurations between two proteins and visualizes them using the cartoon model. The set of green protein instances corresponds to one of the interacting proteins, the colored components represent the second protein in different spatial configurations

Even for the comparison of two configurations, a traditional overlay representation suffers from many occlusion problems and it is hard to perceive the differences between individual solutions. When comparing more configurations, even without a detailed visualization of the hot spot amino acids, the problem becomes even more apparent (Fig. 1).

### Related work

As the selection of the most proteomically relevant PPI configurations is a very challenging task, several algorithms have already been published for re-ranking the configurations according to different criteria. They suggest a subset of configurations that should be explored in detail. As a representative of these attempts, Malhotra et al. [3] presented DockScore, a web server for ranking the individual configurations produced by docking tools. Their idea is based on building a scoring scheme that considers several interface parameters, such as the surface area, hydrophobicity, spatial clustering, etc. This helps the user to reduce the number of configurations to a smaller set, which still has to be explored manually. For this exploration, a visual support is essential, as it enables the user to see the spatial orientation of the contact zones and to compare different configurations. However, DockScore provides only a rudimentary visual representation of top five configurations, which is insufficient for the proper exploration of the configuration space.

Finding a proper visual representation of PPIs can be approached from different perspectives. One technique consists of techniques visualizing the contact zones and their interacting amino acids. The spatial techniques have to address the problem of occlusion and visual clutter caused by the fact that the most interesting parts of interacting proteins, the contact zones, are facing each other inside the configuration. Without transformations or visual enhancements (e.g., through transparency), it is impossible to visually explore the contact zones. Jin et al. [4] presented an open-book view where the interacting proteins are rotated to orient the contact zones towards the camera. The problem with the presented solution lies mainly in the missing information about the interacting amino acids and the unified coloring of the contact zones. An alternative approach presented by Lee and Varshney [5] computes and visualizes the intermolecular negative volume and the area of the docking site. This way the users can observe the volume between the interacting proteins without the need to display the contact zones themselves. This can serve proteomic experts as an interactive tool for studying possible docking configurations, but it does not support their comparison. Similar approaches suggest the construction of an interface surface between the interacting proteins [6, 7]. The surface is visualized as a 3D mesh, encoding the information about the core and peripheral regions from the interface. However, this method also does not support the comparison of multiple configurations.

**Fig. 2:**
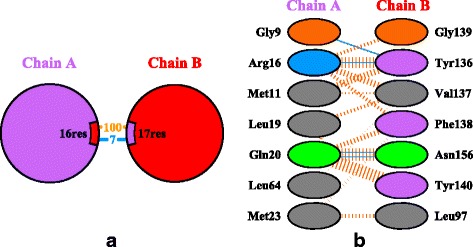
NSE1-NSE3 complex representation in PDBsum. Two abstracted visualizations of the NSE1-NSE3 complex with PDB ID 3NW0 available in the PDBsum database. **a** Overview representation showing the number of amino acids in the contact zones and the types of interactions. **b** Part of the list of interacting amino acids along with individual interactions and their strength. Images taken from the PDBsum database [8]

Two-dimensional abstract representations are also commonly used for the visualization of contact zones, such as the schematic representation used by the PDBsum database [8] (Fig. 2). In the overview visualization, each of the interacting proteins is represented by a circle equipped with information about the number of amino acids forming the contact zones and the number of different types of interactions in-between (e.g., salt bridges, disulphide bonds, hydrogen bonds, or non-bonded contacts). The detailed visualization in PDBsum lists all the contact zone amino acids. The interactions are visualized by lines of different color and thickness, which represent the type and strength of the interactions, respectively. This approach gives a comprehensible overview of one configuration, but comparing it with another configuration is not possible.

Lex et al. [9] proposed a visual analysis tool for the exploration of large scale heterogeneous genomics data for the characterization of cancer subtypes. They use multiple views of the complex data, and one of them is a method for the comparison of different datasets. The abstract representation shows the similarities in the datasets by connecting corresponding blocks of data. The thickness of a connection denotes the degree of similarity. This representation serves well for comparison, but it lacks detailed information about the individual items.

In this paper, we present a systemic tool, COZOID, comprised of a set of methods for the visualization, comparison, and selection of numerous docking configurations. The combination of our proposed methods eliminates the problems associated with the existing solutions and provides proteomic experts with an intuitive and user-friendly tool for the interactive exploration of PPIs. Our tool is integrated into the CAVER Analyst software [10], which allows for the analysis and visualization of biomolecules, and therefore, contains many relevant features, such as different molecular visualization modes, measurement tools, etc. The input PPI configurations are provided by the existing computational tools and our solution is designed for dealing specifically with a large number of configurations.

## Methods

### COZOID overview

Our newly proposed system enables for the efficient visual exploration of a large number of PPI complexes. For a better understanding, we introduced the following notation. A protein *P* consists of a set of amino acids forming a polypeptidic chain. A complex *C* is represented by a set of mutually interacting proteins. In our case, we focus primarily on the interactions between two protein structures *P*_1_ and *P*_2_, which form a complex *C*(*P*_1_,*P*_2_). The mutual spatial orientation of the interacting proteins in the complex forms a configuration. The *i*-th configuration of complex *C*(*P*_1_,*P*_2_), denoted as *CONF*_*i*_(*C*(*P*_1_,*P*_2_)), represents one of the possible mutual orientations of this complex. Generally, there can be *n* (1≤*i*≤*n*) possible configurations for a given complex, and the task is to select the configuration that is the most relevant one from a proteomics point of view. The decision is based on various pieces of knowledge about the geometric arrangement of the configuration as well as other aspects, such as knowledge of the contacts between the amino acids present in the contact zone of the given configuration. Therefore, the selection of the most relevant configurations cannot be completed automatically and requires insights from the proteomic expert. This represents a typical domain-related problem, which has to be supported by specifically designed visualizations.

**Fig. 3:**
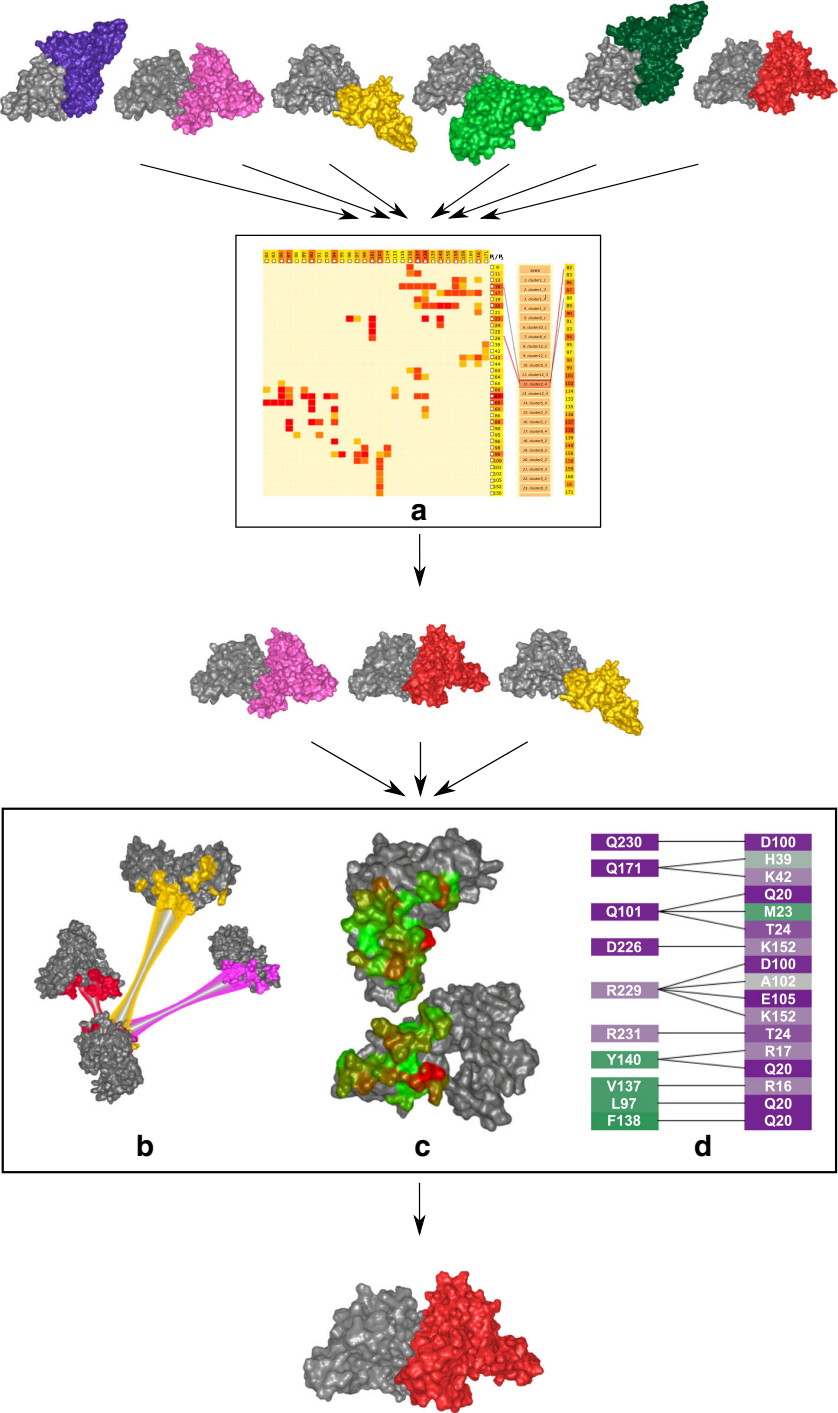
Workflow overview. The exploration process followed by the domain experts and our proposed supporting visualizations. **a** The Matrix view represents an overview of all input configurations, obtained by one of the existing computational tools. **b** The Exploded view enables the user to explore the contact zones and their differences for a set of selected configurations. **c** The Open-Book view animates the opening of a selected configuration. **d** The Contact-Zone list-view supports the detailed comparison of the constitution of the contact zones of selected configurations

The visualization methods proposed in this paper allow the user to visually explore a set of possible configurations detected by one of the existing computational tools and to select the most proteomically relevant ones. The users have to iteratively filter out those configurations that do not fulfill the given specific criteria. The proteomic expert workflow, along with our proposed visual support of its individual stages, is depicted in Fig. 3. The input datasets, consisting of dozens of configurations between two interacting proteins, were computed using the HADDOCK [11] and PyDock [12] tools. However, any of the existing tools for protein-protein docking can serve as a source of input data for our system.

The proposed visualizations are based on the precondition that the users already have initial knowledge about the interacting proteins. Thus, the experts are able to define a pair of amino acids that are expected to interact. This is not restrictive, as computational tools also require this information to produce a meaningful set of configurations. In other words, we are using similar input information as the computational tools. The second possibility is that the users do not have this information but are aware of an already explored protein complex with a similar structure that can serve as a reference (primary) complex for further comparison and exploration. In this case, the computational tools usually produce even more configurations, but most of them are irrelevant and have to be filtered out. Our tool can utilize the information about the interactions in the primary complex and enhance the filtering process.

Our methods have been designed specifically to help proteomic experts answer the following questions:

- Q1: Which configurations contain a selected interacting pair of amino acids (and what is the frequency of the occurrence of this pair in all configurations)?
- Q2: Which pairs of amino acids are present in a given configuration?
- Q3: How close are the amino acids in the contact zone and which are the closest ones?
- Q4: How similar and different are the contact zones in the configurations?
- Q5: What are the physico-chemical properties of the amino acids in the contact zone?
- Q6: What are the differences between the sets of amino acids in the contact zones of different configurations?

Answering these questions helps the proteomic experts to better understand the interactions in the protein-protein complexes and to evaluate the correctness of the given configurations. The proposed visualizations enable one to find the answers by interactively exploring the configurations which is demonstrated in the supplementary video as well (see Additional file 1). In the following chapters, we introduce our proposed views in detail.

### Matrix view

**Fig. 4:**
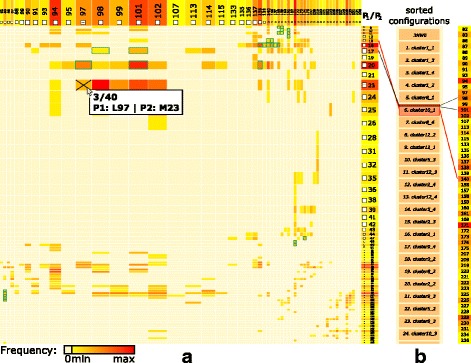
Matrix view for the exploration and filtering of the input configurations. **a** Matrix view showing the aggregated information about the presence of mutually interacting amino acids in all configurations. Horizontal and vertical axes contain the lists of amino acids in the contact zones of the interacting proteins *P*_1_ and *P*_2_. **b** The side view shows individual configurations sorted according to their similarity to the primary configuration. The interaction with the side view enables to gain more detailed information about the configurations and their interacting amino acids. The central part of the side view consists of a scrollable list of individual configurations. The vertical list of amino acids (the rightmost column) is the same list as the one on the horizontal axis. The configuration in focus contains one polyline connecting those two amino acids from the contact zone which are the closest ones (red lines). The remaining interactions between amino acids are marked with black polylines. The green borders of some matrix cells represent the pairs which are present in the configuration selected in the side view. The selected cells are marked with a cross. It is possible to enlarge a selected row and column using an interactive lens

When using a computational tool to generate possible configurations, the resulting set *S*={*CONF*_*i*_(*C*(*P*_1_,*P*_2_));1≤*i*≤*n*}, *n* can be very large, ranging from dozens to hundreds. This amount is impossible to explore manually; thus, some preliminary filtering is crucial. The filtering stage is designed to answer question Q1. We propose a matrix-based visualization inspired by commonly used heat maps (Fig. 4a). The rows and columns in the Matrix view correspond to the interacting proteins *P*_1_ and *P*_2_, respectively. Each row or column represents one amino acid present in a contact zone in some of the configurations *CONF*_*i*_(*C*(*P*_1_,*P*_2_)). The rows and columns are formed only by those amino acids from the interacting proteins that are in contact in at least one configuration. The contact between the amino acids is based on their Euclidean distance. Two amino acids are considered to be in contact if their distance is between 3 and 5 Å. This range can be interactively changed by the user. The color of each cell in the matrix corresponds to the number of occurrences of the corresponding interacting amino acids in the set *S* of all configurations. The colored lists of amino acids can be interpreted as histograms, encoding the number of their occurrences. The intense red color represents the pairs of amino acids that are interacting in most of the configurations. The Matrix view serves directly for filtering out improbable solutions using the interactive user-driven selection of cells. The selection is performed by clicking on individual cells. Moreover, the matrix allows the expert to selecSut a combination of several pairs of amino acids. This is useful if the user wants to further explore only those configurations that contain specific interactions, such as between the amino acid pair *A*, *B* and simultaneously the pair *C*, *D*.

The big advantage of the Matrix view is its independence from the size of the input set of possible configurations. The number of rows and columns is limited by the size of the interacting proteins, meaning that in the worst case, it corresponds to the total number of amino acids in these proteins. However, in most cases, the number of amino acids in the contact zones is much smaller than the total number of amino acids. Each configuration of the input dataset then increases the counters in the respective matrix cells. In the case of many interacting amino acids, the cells in the matrix can become too small. In these situations, the users can employ the table lens technique introduced by Rao and Card [13], which can be applied to both rows and columns in the matrix (Fig. 4a).

To provide the users with more detailed information about individual configurations, the Matrix view contains an additional side view, which is positioned directly next to the matrix (Fig. 4b). The user can select a primary configuration to which all the remaining configurations are compared. An example of a primary configuration can be a crystal structure downloaded from the PDB database. We propose the following ranking score, which indicates the similarity between the contact zone of a given configuration and the primary configuration. One of the interacting proteins, e.g., *P*_1_, is selected as a reference protein, while the second protein, e.g., *P*_2_, is marked as the paired protein. The score is computed in the following way.

- For each match of an amino acid in the contact zones from the reference proteins of the compared and the primary configuration, the similarity score is increased by one.
- For each matching interaction pair in the contact zones from the compared and the primary configuration, the similarity score is increased by four.
- For each missing interaction pair in the contact zones from the compared and the primary configuration, the similarity score is decreased by one.

This score was determined experimentally while designing and testing the view (see Results chapter). The central part of the side view consists of a scrollable list of individual configurations from a subset of *S* that was filtered with the Matrix view. The configurations are ordered according to their similarity scores, from the most similar to the least similar ones. The primary configuration is always displayed as the first one on the top of the list.

The side view helps to answer questions Q2 and Q3, as it enables an iterative search through the list of configurations and the exploration of all pairs of interacting amino acids for each configuration. The user can select a configuration to focus on by clicking on it. By default, each configuration in focus contains one polyline connecting two amino acids from the contact zone that are the closest among all the possible pairs (Fig. 4b). The user can hover the mouse over the lists of amino acids on the left and right side and inspect the corresponding connection lines for a given amino acid. By clicking on the rectangle representing a given amino acid, the connection lines remain in the view. The pairs of amino acids that form the configuration in focus can be highlighted in the matrix (with green border rectangles in Fig. 4a). From the color of the matrix cells, the user can immediately estimate the number of configurations in which these pairs are present. Vice versa, by interacting with the matrix and selecting the given rectangles, the side view is automatically filtered to show only those configurations that satisfy the filtering condition.

The Matrix view serves as the first filtration tool for selecting only those configurations that contain a desired combination of interacting amino acids. This filtering cannot be automated because the frequency of a given pair in configurations does not correlate with the importance of these configurations. The most frequent pair of interacting amino acids can be of the same interest as a pair interacting only in one configuration. Therefore, insights from the proteomic expert in combination with the interaction possibilities from the Matrix view have proven to be a very efficient and powerful solution. Selected configurations can be further processed by the following visualization methods.

### Exploded view

**Fig. 5:**
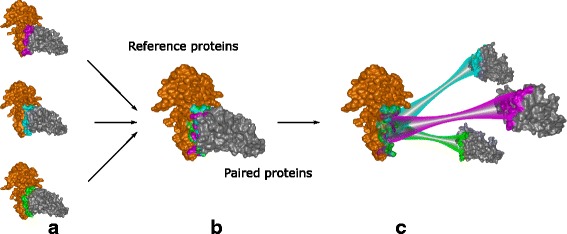
Exploded view. **a** Three configurations represented by surfaces with highlighted contact zones. **b** Aligned configurations. Their contact zones are almost completely occluded. **c** Exploded view of these configurations. A different color is used for each contact zone

The proteomics experts are already familiar with the manipulation of molecules in a three-dimensional (3D) environment; thus, a 3D representation has to be an integral part of the workflow. Moreover, the 3D space helps to find answers for questions Q3-Q5, which are related to the appearance of the contact zones of selected configurations and the properties of interacting amino acids (expressed by different coloring schemes). Exploring and comparing many structures in 3D at once suffers from problems such as high overlap, occlusion, and visual clutter (Fig. 5b). Traditionally used spatial representations are not sufficient. To overcome these limitations, we adapted an exploded-view technique, to enlarge the distance between the interacting proteins. Figure 5c shows the comparison of three configurations using our proposed Exploded view.

The main principle of the Exploded view is the following. First, all the reference proteins taken from the configurations selected in the Matrix view are aligned using the Combinatorial Extensions from the structural-alignment algorithm [14] so that their 3D spatial representations overlap (Fig. 5). Here, it is important to understand that the reference protein shown in Fig. 5b (the brown one) actually represents three overlapping aligned reference proteins, each coming from one configuration. The set of paired proteins interacting with the reference proteins is positioned around the aligned reference proteins with an enlarged distance.

To ensure that the paired proteins in the Exploded view will not collide with each other, we arrange the paired proteins into a parabolic regular grid. For each reference protein and it’s paired protein, the Exploded view retains the information about their interaction. If several configurations are exploded at once, the Exploded view contains many paired proteins arranged around the aligned reference proteins. As the change in the position of the exploded proteins can cause disorientation in the scene, the pairing information between the corresponding reference proteins (aligned) and paired proteins (“exploded”) is initially indicated as a partially transparent tube that connects the centers of their contact zones. The radius of the tube is modulated (it is smaller in the middle of the tube to reduce the visual clutter). Once the user understands the overview of the protein spatial arrangement, the tube can be switched off. The pairing information is also encoded by color (a different color is used for each configuration). If the contact zones contain colliding amino acids (i.e., their mutual distance is less than 3 Å), the residues are indicated by a red color.

Figure 5 depicts a set of three configurations before (a, b) and after (c) applying the Exploded view. The Exploded view removes the problem of overlapping paired proteins. It also helps to see the shape and position of the contact zones. However, this solution does not solve the problem where the contact zones face each other, meaning that the user has to adjust the camera to observe the contact zones of the reference and paired proteins from a perpendicular viewing direction. This manipulation does not enable the user to see both contact zones simultaneously. This problem is solved by the proposed Open-Book view, which is presented in the following section.

### Open-Book view

The Exploded view does not allow one to observe both parts of a given contact zone simultaneously. The proposed Open-Book view is designed to specifically answer questions similar to Q5, which addresses a detailed exploration of one selected contact zone in the complex *C*(*P*_1_,*P*_2_). This involves the presentation of the information about different properties of individual amino acids forming the contact zone and their pairing.

**Fig. 6:**
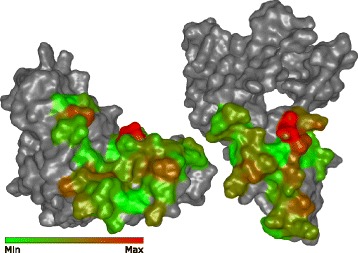
Open-Book view. Open-Book view enables the user to explore the contact zones between the interacting proteins simultaneously. On the left there is the reference protein and on the right there is the corresponding paired protein. The surface of the contact zones can be color-coded according to different criteria. Here the color represents the distance between the pairs of amino acids (red represents the closest ones, green the most distant ones)

The Open-Book view is activated if the user selects one of the configurations from the Exploded view. The selection is performed by clicking on the connection tube from the desired configuration *CONF*_*i*_(*C*(*P*_1_,*P*_2_)) in the Exploded view. The other configurations are automatically hidden, the selected configuration returns to its initial position (before applying the Exploded view), and an animated transition for the opening of *CONF*_*i*_(*C*(*P*_1_,*P*_2_)) is launched. When animating the opening, the reference and paired proteins are rotated and translated so that they are positioned next to each other and the contact zones are facing towards the observer (see Fig. 6).

The algorithm performing the opening computes the vectors defining the orientation of the contact zones (their normal vectors). From the normal vectors and the camera position, we compute the rotation angle, which is then applied to the reference and paired protein. To maintain the information about the amino acid pairings, the user can also visualize individual connections between these pairs through simple lines.

The contact zones represented by their surfaces can be color-coded according to multiple criteria. The color can encode the distance between the amino acids or represents different physico-chemical properties of the amino acids or their atoms, such as hydrophobicity or partial charges. The coloring scheme used in the Matrix view represents the so-called conservation of the amino acids in all configurations. It can also be used to color the contact zone. The surfaces can be augmented with labels to inform the users about the type and identifier of individual amino acids.

In both the Exploded view and the Open-Book view, a protein can also be represented by other traditionally used visualization styles, such as cartoon, spheres, balls&sticks, sticks, etc. Moreover, these methods can be combined. For example, the proteins can be represented by the cartoon style and the amino acids in the contact zones can be visualized using the sticks representation to see their spatial orientation.

If the task is to compare individual configurations with respect to the pairs of interacting amino acids, a further drill-down is necessary. Therefore, in the next section, we propose another abstract view supporting mainly the comparison of paired amino acids in individual contact zones from selected configurations.

### Contact-Zone list-view

**Fig. 7:**
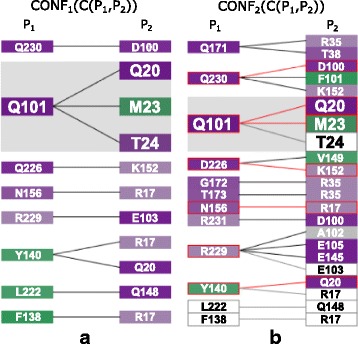
Contact-Zone list-view. This view shows the comparison of one configuration, the primary one (**a**), with another selected configuration (**b**). For better comparison of configurations, the corresponding amino acids are interactively highlighted by zooming in. The view is sorted (and colored) according to hydrophobicity of the amino acids in the *P*_1_ protein. Red color indicates the matches between the contact zone amino acids of the primary and the compared configuration. White rectangles indicate amino acids that are present in the primary configuration but are missing in the compared one

The Contact-Zone list-view helps to answer questions related to the comparison of the contact zones at the level of the individual amino acids, such as in Q6. The list for one configuration consists of two sets of amino acids in the contact zones, each set coming from one interacting protein (see Fig. 7). The left part of the view contains all amino acids coming by default from the reference protein, while the right part is formed by their interaction counterparts in the paired protein. However, the order of proteins in the list-view can be changed. The order depends on the current task, i.e., if we want to compare the constitution of contact zones from the reference or the paired protein in the given configurations. The view contains all possible connections (with respect to the distance) between the amino acids from both contact zones. To avoid the intersection of lines representing the connections, some amino acids on the right side are repeated – one instance for each reference protein amino acid within a user-defined distance. This solution was adopted because without these repetitions, there would be many line intersections, which substantially decreases the readability of the representation (see Fig. 2b).

**Fig. 8:**
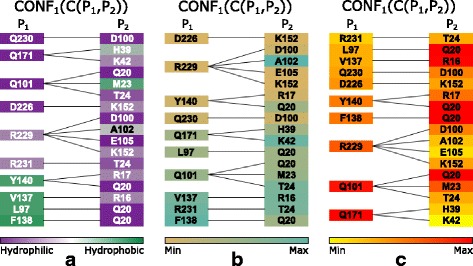
The Contact-Zone list-view and different properties. Sorting of the Contact-Zone list-view according to different properties of amino acids – **a** hydrophobicity, **b** mutual distance, **c** frequency of occurrence of the pairs in all configurations

For each configuration, one list-view is created and all the list-views are juxtapositioned so the user can see and visually compare the constitution of the contact zones from all selected configurations. The user can modify this representation by changing the color, which can encode different properties for the amino acids mapped onto their corresponding rectangles. The properties are the same as those mapped onto the surface of the contact zone in the Exploded and Open-Book views. The left part of the list can then be sorted according to these properties (see Fig. 8). Moreover, by clicking on individual rectangles representing the amino acids, the corresponding amino acids are selected in the 3D view as well.

The principle steps for building the Contact-Zone list-view are the following. For all configurations, which should be visualized in the Contact-Zone list-view, we find the interacting pairs of amino acids in their contact zones.

Then, the list of amino acids present in all reference proteins from the selected configurations is created. Now, for each configuration, we take the interacting amino acids from the paired proteins, sort them according to a selected criterion (e.g., hydrophobicity), and add them to the Contact-Zone list-view. The amino acids in the left part of the Contact-Zone list-view are always sorted in the same way for all depicted configurations. Similar to the Matrix view, the user can select a primary configuration to which all the remaining configurations are compared (see Fig. 7b) using the proposed ranking score algorithm, which is described in “Matrix view” section. The Contact-Zone list plots the configurations ordered from left to right by the similarity score from the most similar to the least similar. The Contact-Zone list-view of the primary configuration is always displayed as the first one from the left side of the view.

The user can select between two visualization modes – the *compare* and the *compact* list-view. In *compare* mode, the amino acids in the contact zone in the primary configuration that are not present in the contact zone from any other configuration are depicted as white rectangles with labels giving the names of the missing amino acids (see Fig. 7b). The *compact* mode omits these missing amino acids to save space. In both modes, the matches between amino acids in the primary configuration are highlighted with red bordered rectangles and connecting lines. This way, the user can immediately see which amino acids are present in both the primary configuration as well as the other configurations and which amino acids are missing. To guide the visual comparison, we also introduced interactive highlighting and, if necessary, zooming to corresponding amino acids in different configurations.

## Results and discussion

To demonstrate the usability of our proposed techniques, we selected three representative basic types of PPI patterns present in SMC complexes [15]. SMC (Structure Maintenance of Chromosome) complexes are the key players in chromatin organization where they ensure the stability and dynamics of chromosomes. The way the subunits of these complexes interact with each other is key for their functions [16]. A visual representation of such information is highly beneficial as it helps to reveal the spatial relationships between the subunits in an intuitive way. The three basic PPI types are coiled-coil, pocket-string, and surface-surface interactions [17]. In the following subsections, we demonstrate the usefulness of our proposed visualizations on these three types of interactions.

### Surface-surface interaction

The most frequent surface-surface interaction type was tested on the NSE1 and NSE3 proteins in the SMC5/6 complex. This interaction has been analysed as it represents a dimer of kite proteins, which are critical for the function of eukaryotic SMC5/6 and bacterial SMC complexes [15, 18, 19].

The crystal structure of the human NSE1-NSE3 dimer was already examined in detail and the resulting configuration is already published in the PDBsum database under the PDB identifier 3NW0. Therefore, it can serve as a primary testing complex for both the computational tools as well as for our proposed visualizations. To restrict the set of possible docking configurations, we selected the web version of the HADDOCK tool and a pair of interacting amino acids, i.e., methionine with ID 23 from the reference protein and leucine with ID 97 from the paired protein (Fig. 2b). This selection was based on experimental data from previous works [19–22]. The HADDOCK analysis resulted in 40 possible configurations. HADDOCK groups the configurations into clusters, according to their similarity, which is defined internally by the HADDOCK score. In our case, it led to 10 clusters each containing 4 configurations.

The computed configurations were loaded into our COZOID visualization system, which interactively links all the proposed visualizations. From these configurations, the Matrix view was computed first, which contains the frequencies of all the pairs of amino acids within the interaction distance within these 40 configurations. The matrix identified configurations containing pairs of interacting amino acids with interaction distances smaller than 4 Å. In our particular case, the leucine 97 and methionine 23 amino acids were within this interaction distance in only three configurations out of the initial 40 (Fig. 4). The Matrix view helped to filter these immediately through a simple interaction with the view. The remaining 37 configurations were automatically hidden in the remaining views.

**Fig. 9:**
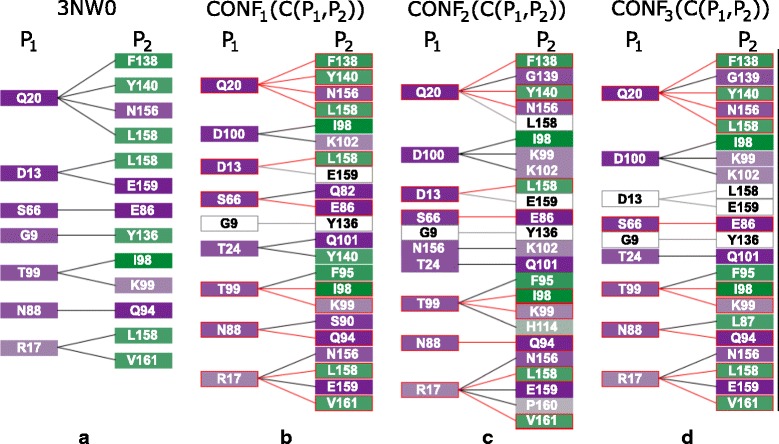
Surface-Surface Interaction – best HADDOCK configurations. Example of four configurations represented by the juxtapositioned Contact-Zone list-view. **a** Primary 3NW0 crystal structure, **b**, **c**, **d** three selected best-fit HADDOCK models. The lists are colored and sorted according to the hydrophobicity of the amino acids in the reference protein in each selected configuration

In the next step, we switched to the Contact-Zone list-view and compared the list of amino acids from the 3NW0 crystal structure with the lists of all three selected configurations. Figure 9 shows the comparison between the 3NW0 structure and the three selected HADDOCK configurations. From the given portion of the Contact-Zone list-view, the similarities and differences between the 3NW0 crystal (in the leftmost list) and the three selected HADDOCK configurations at the level of the individual amino acids are clearly visible. Additionally, the pairs of the interacting amino acids identical to the 3NW0 crystal structure are highlighted (red lines in Fig. 9). The left-to-right order of the modelled configurations in Fig. 9 reflects their similarity to the primary crystal structure, based on the number of identical pairs of amino acids (the best model is next to the crystal).

Finally, the 3NW0 crystal and three selected configurations were explored using the 3D representations with the aim of exploring the constitution, mutual distances, and properties of the contact zones in detail. In 3NW0, the first NSE1 interacting protein was selected as the reference protein and all three configurations were aligned with respect to the paired proteins. The paired proteins were positioned around the reference one. Figure 5a shows the situation where the three selected configurations are visualized using a commonly available method. The configurations are represented as surfaces and the contact zones are highlighted using different colors. However, the most interesting parts, i.e., the contact zones, are hidden (Fig. 5b).

Our Exploded view overcomes this limitation so the individual contact zones from all the paired proteins are clearly visible (Fig. 5c). Moreover, if we point the camera towards the aligned reference proteins, the differences between the positions in the contact zones in the reference proteins can be observed as well. The Exploded view representation gave us the information about the mutual positioning of the individual configurations with respect to the positions of the contact zones.

Using our tool, the investigation can go even deeper to the level where individual contact zones can be explored in detail using the Open-Book view. By animating the opening of the protein complex, we were able to look inside the contact zone. The Open-Book view enhancements, i.e., labelling the surface of the contact zones with the names of the corresponding amino acids and coloring them according to different properties, were highly beneficial for exploring the physico-chemical and geometric properties of the individual amino acids.

### Coiled-coil interaction

For the second type of interaction, we picked the SMC3 coiled-coil arm from the SMC complex [16]. The interaction site is formed by two helical fragments from the SMC3 protein. The primary structure is published under the PDB identifier 4UX3 [23].

**Fig. 10:**
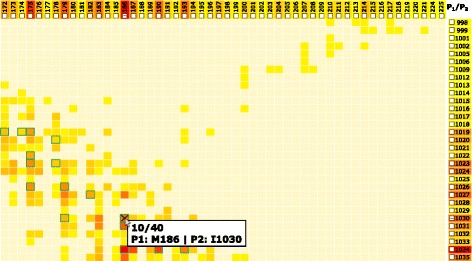
Coiled-Coil Interaction – the Matrix view of interacting amino acids in all HADDOCK models. The Matrix view indicates that the selected pair of M186 and I1030 amino acids is present in 10 out of 40 loaded models

Using this structure, the results of both the HADDOCK and the PyDock tools were tested. The HADDOCK results contained 40 output configurations. Using the Matrix view, we set the interaction distance threshold between 3 and 5 Å and selected methionine 186 and isoleucine 1030 as the initial pair of interacting amino acids (Fig. 10). These amino acids were used as the input restraints for the HADDOCK computation as well. These restraints were applied to select the correct configurations in the Matrix view (Fig. 10).

**Fig. 11:**
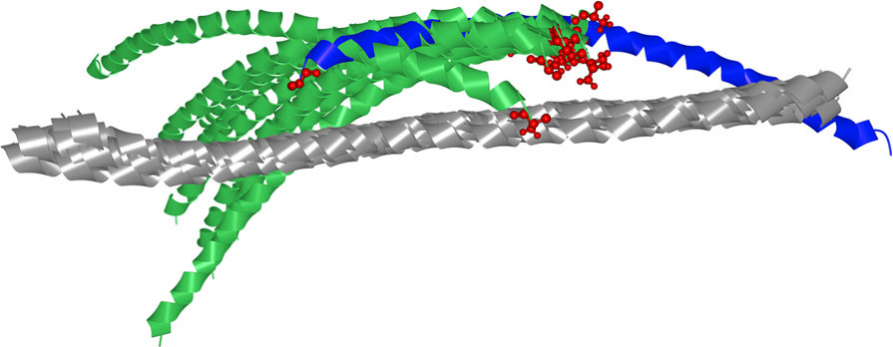
Coiled-Coil Interaction – 4UX3 crystal (blue) and 10 selected HADDOCK configurations (green). The first A172 amino acid (red) is highlighted in all loaded structures. The opposite orientation of 4UX3 and HADDOCK models is clearly visible

Next, the selected configurations were structurally aligned to the primary 4UX3 structure in 3D space. Afterwards, we selected the first amino acid (A172) within the respective helices and visually compared their positions in the 3D view. In this case, it was not even necessary to use other views to see that the preselected HADDOCK configurations exhibited a wrong orientation of the aligned helices. In all the output models, the A172 amino acids were located on the opposite side in comparison with the primary 4UX3 crystal (see Fig. 11). The 3D view from COZOID helped to reveal this misorientation intuitively and quickly, without a detailed exploration of the HADDOCK configurations one-by-one.

**Fig. 12:**
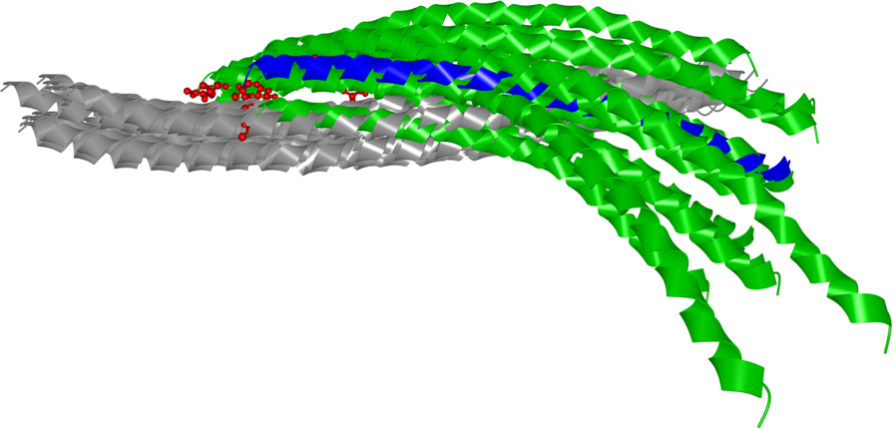
Coiled-Coil Interaction – 4UX3 crystal (blue) and 14 selected PyDock configurations (green). In these PyDock configurations, all A172 amino acids (red balls and sticks) are positioned at the same side as in the crystal structure (blue)

As for the PyDock results, 28 out of 100 output PyDock models were selected using the Matrix view; the M186 and I1030 interaction pair was used to filter the results. The visual selection (based on A172 position judgement) provided us with 14 models in the correct orientation (see Fig. 12).

**Fig. 13:**
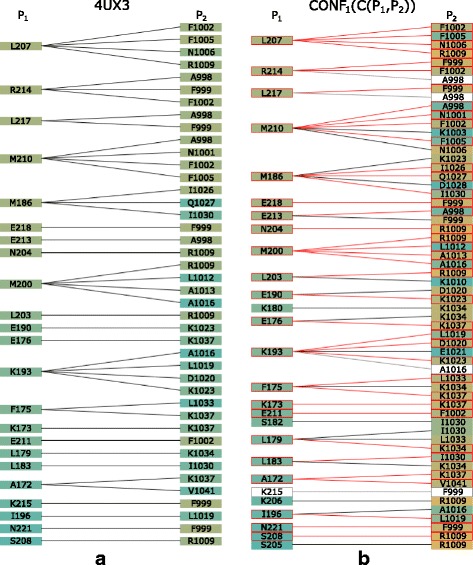
Coiled-Coil Interaction – best fitting PyDock configuration. Contact-Zone list comparing the **a** 4UX3 crystal with **b** one best fit PyDock model with respect to the distances of amino acids

**Fig. 14:**
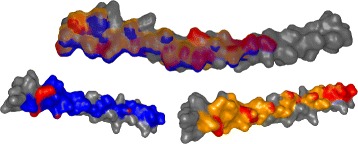
Coiled-Coil Interaction – best fitting PyDock configuration. The Exploded view showing the contact zone of the best fitting PyDock model (orange) and the 4UX3 crystal (blue). On the top, the overlapping contact zones on the reference protein are shown. The bottom part of the image depicts the paired proteins

In the final step, we compared the Contact-Zone lists of the selected models with the original crystal structure (4UX3). Figure 13 shows the similarities (highlighted in red) of one of the selected models to the crystal. It is the best model, and fits the crystal structure very well. The Exploded view comparison of the contact zone from the crystal structure and the selected model can be observed in Fig. 14.

### Pocket-string interaction

**Fig. 15:**
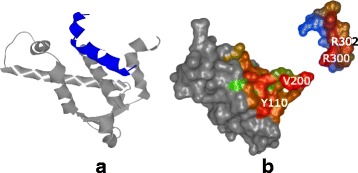
Pocket-String Interaction – 3EUH crystal structure. **a** 3EUH crystal structure consisting of the domain containing the pocket (grey) and the helical fragment of the second domain (blue), shown using the cartoon representation. **b** The same structure shown with the Open-Book view. The contact zones are colored according to the distance between the interacting amino acids and the labels of the two closest pairs are shown

**Fig. 16:**
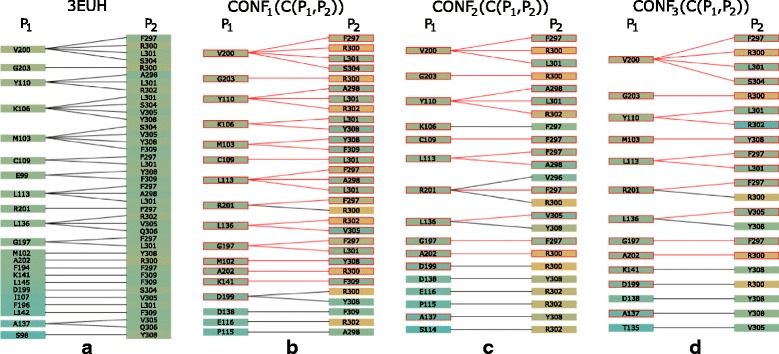
Pocket-String Interaction – best HADDOCK configurations. Contact-Zone lists of the selected HADDOCK configurations sorted according to the distance of the amino acids. **a** The primary 3EUH crystal structure, **b**, **c**, **d** three selected HADDOCK models. The sorting shows that the V200-R300 pair is one of the closest ones in the crystal as well as in all selected models

For the pocket-string interaction type, we selected an interaction present in the crystal structure from the MukE-MukF complex (PDB identifier 3EUH) [24]. The pocket is formed by the winged helix domain of the MukE protein, while one of the MukF helical fragments is sitting inside the MukE pocket (Fig. 15a). This time, we selected valine 200 and arginine 300 as the pair of amino acids for the docking restraints. These were the closest contact amino acids in the structure, as can be observed from the Contact-Zone list ordered by the distance of the interacting amino acids (see Fig. 16), as well as from the Open-Book view of the crystal structure (Fig. 15b).

**Fig. 17:**
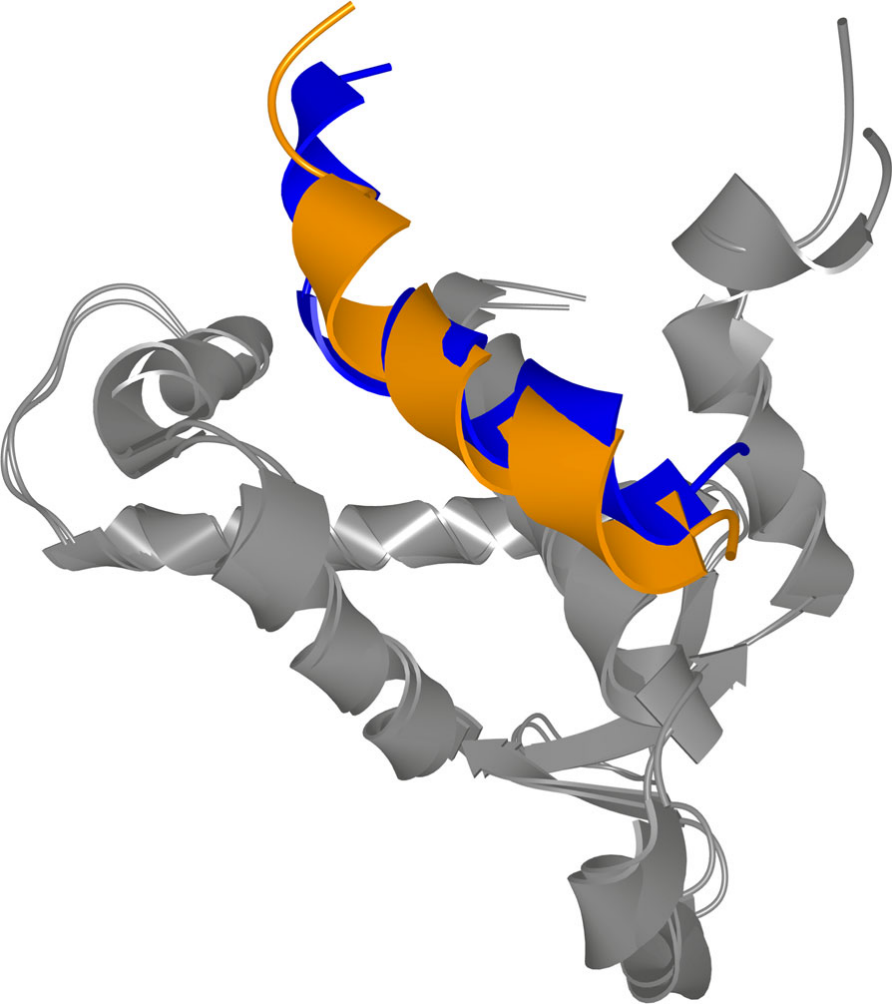
Pocket-String Interaction – the best fitting HADDOCK configuration. The best fit HADDOCK configuration (orange) aligned with the 3EUH crystal structure (blue)

The docking models were again generated with both HADDOCK and PyDock docking tools. The HADDOCK run resulted in 32 output configurations, which were first scrutinized using the Matrix view, using the initial V200-R300 amino acid pair. This first selection step filtered away only 8 models, leaving 24 models for further analysis. Then, we repeated the Matrix view filtering using the second tightest amino acid contact in the crystal (tyrosine 110 and arginine 302) (Fig. 15b). This filtration resulted in 6 docking models. The Contact-Zone lists of these models were compared with the original crystal structure (3EUH), resulting in an ordered list of the best models (Fig. 16). The visual exploration confirmed that the first model from the Contact-Zone list fits best to the original structure (Fig. 17).

PyDock docking provided 100 models, which were analysed similarly to the HADDOCK models. The selection steps with the Matrix view, including the first filtration step with the initial amino acid pair and the filtering with the second amino acid pair, resulted in 32 and 19 models, respectively. The Contact-Zone lists of these models were then compared with the original crystal structure. The models most closely matching the original crystal structure, which was detected using the Contact-Zone list, were then visually explored in 3D using the Exploded view and the Open-Book view. This step revealed that the best five models from the list are very close to the original crystal, though none of them precisely fits the crystal structure.

**Fig. 18:**
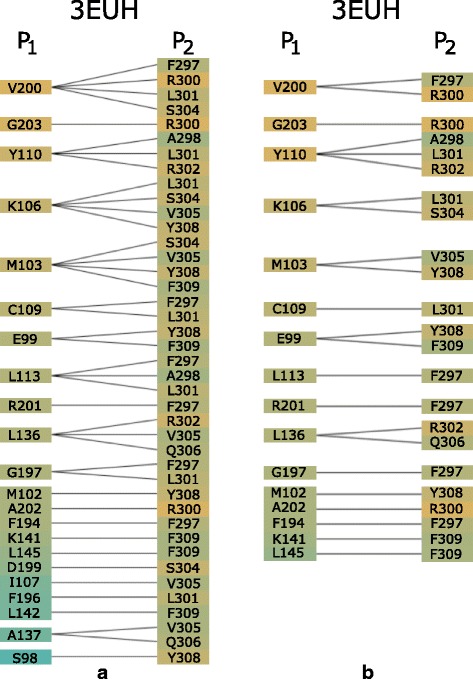
Contact-Zone lists of 3EUH crystal structure computed with different distance parameter settings. **a** Contacts computed with the distance parameter 5 Å. **b** Contacts computed with the distance parameter 4 Å. The Contact-Zone lists are sorted according to the mutual distance of the amino acids

**Fig. 19:**
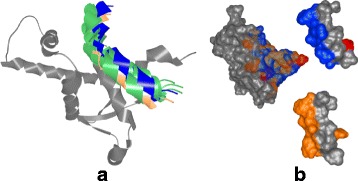
Pocket-String Interaction – the best fitting PyDock configurations. **a** The best 5 PyDock configurations with the distance parameter 4 Å, aligned with the 3EUH crystal structure (blue). The configuration, which exhibited the most similar contacts with the crystal, is orange and the remaining configurations are green. **b** The Exploded view showing the comparison of the contact zones of the best PyDock configuration (orange) with the 3EUH crystal structure (blue)

Here, we took the advantage of our testing setup (using the tightest contacts between the interacting amino acids) and altered the interaction distance parameter in the Matrix view for the selection procedure. All PyDOCK models were re-evaluated with the distance parameter set to 4 Å (compared to the previous 5 Å default parameter settings). As expected, fewer configurations containing the V200-R300 and Y110-R302 amino acid pairs were found within the 4 Å distance (the Matrix view selection steps resulted in 21 and 13 models, respectively). However, the altered distance parameter also resulted in a different ranking of the configurations in the Contact-Zone lists. Figure 18 shows the comparison of the Contact-Zone lists for the 3EUH crystal structure computed with 5 Å and 4 Å distance parameter settings. It can be seen that the decreased distance parameter eliminated several amino acid pairs with distance greater than 4 Å from the crystal structure Contact-Zone list. The eliminated pairs were not considered in the new Contact-Zone list ranking, where five models, the most similar to the crystal, were once again selected (Fig. 19a). Four of these five models overlapped with the five best models detected with the previous system set-up; however, a new model with a closer match was also identified (Fig. 19b).

This test indicates the robustness of our tool with different parameter settings and its potential for experimental use in proteomics. Our tool can also be used to select an alternative input pair of interacting amino acids, which then serves as the input for the computational tools. These amino acids might be selected based on the COZOID analysis of the 3NW0 crystal (using the Matrix view or Exploded view) when searching for the most central and closest amino acids.

Altogether, COZOID helped us to quickly select the best docking configuration using several visualization approaches. First, the Matrix view allowed us to pick models containing a particular pair of interacting amino acids. Next, with the Contact-Zone list, we sorted these models based on the similarity of their contact zones with the original crystal structure. Using the 3D Exploded view, the best model was determined and confirmed. While the Exploded view is already available in some of current 3D visualization tools, the power of its combination with our other proposed approaches lies in the speed, user-friendly design, and highly interactive selection mechanism. Additionally, a similar workflow can be applied for the selection of docking models from homologous proteins, which is not available in the PDB database, yet is often used when different model organisms are employed in proteomic studies.

For example, our Contact-Zone list can be used in the experimental design of mutants by replacing key contact residues. This tool can be used by proteomic expert to select amino acids in the contact zones that could be mutated, i.e., replaced by other amino acids. The ultimate goal of these mutations could be to strengthen the interactions in the contact zone or, completely destroy the interaction between the involved proteins.

## Conclusions

In this paper, we have presented COZOID, a new tool for the visual exploration of configurations of two interacting proteins. It introduces a set of visualization methods for the exploration and evaluation of proteomic relevance of large sets of configurations detected with existing computational tools. Our proposed methods were designed to follow and support the workflow followed by proteomic experts. We described the design rationale and the principles of these methods, as well as their linking and interaction possibilities. We tested these methods on real datasets of the SMC complex subunits and demonstrated their usability in three studies covering the most common interaction types. Our aim was to overcome the drawbacks of the existing methods for visual analysis and comparison of configurations, which provide users with traditional 3D view and exploration of individual configurations one-by-one. Additionally, specialized techniques enabling to explore the content of the contact zone are completely missing. Therefore, our proposed solution provides proteomic experts with information that is very hard or even impossible to obtain using these previously available methods. The system enables iterative filtering of the configurations that do not satisfy given criteria in the individual stages of the workflow. The executable binary, along with the exemplary dataset and user guide are available in the supplementary material of the manuscript (Additional files 2, 3, and 4).

In the future, we plan to focus on the extension of our proposed techniques in cases where the user has no a priori knowledge about the protein complex, but can still feed in experimental data from mutagenesis or crosslink analysis.

## Additional files

Additional file 1 **Supplementary video.** Video showcasing the software tool in action. (MP4 17,613 kb)

Additional file 2 **Software build.** Executable binary file of the software tool. (ZIP 118,784 kb)

Additional file 3 **Example data.** Testing dataset used in the manuscript. (ZIP 2662 kb)

Additional file 4 **User guide.** User guide for the software tool. (PDF 3502 kb)

## Abbreviations

PPI: Protein-protein interaction
COZOID: Contact zone identifier
SMC: Structure maintenance of chromosome
PDB: Protein data bank
