# Supplementary material for: COZOID: contact zone identifier for visual analysis of protein-protein interactions
Source: BMC Bioinformatics. 2018 Apr 6;19:125. doi: 10.1186/s12859-018-2113-6 (PMC5889581; doi:10.1186/s12859-018-2113-6)

COZOID

User Guide

Introduction **2**
About COZOID 2
Hardware and Software Requirements 2
The Input Data Specifications 2
Launching COZOID 2

User Interface **3**
Main Menu 3
Top Toolbar 4
Main Windows 5
Top left panel 5
Structure Overview 5
Structure Selections 5
Visualization window 6
Right panel 6
Residue Matrix 6
Contact Zone Graphs 8
Coloring 9
Bottom left panel 10
3D View Controls 10
Surface Configuration 11
Bottom panel 11
Structure Sequence 11

Guided Example **12**

Introduction

About COZOID

COZOID (COntact ZOne IDentifier) is a system for the exploration of large sets of protein-protein complexes in a fast and intuitive way.
It was developed in cooperation with:
Human Computer Interaction Laboratory, Faculty of Informatics,
National Centre for Biomolecular Research,
and Central European Institute of Technology,
Masaryk University, Brno, Czech Republic.
COZOID software is based on CAVER Analyst tool (www.caver.cz).

Hardware and Software Requirements

Minimal configuration:

- Java 1.7
- 1 GB RAM
- 32-bit architecture
- Intel HD family graphics adapter
- Microsoft Windows 7,8,10 (Linux or Mac OS X is only partially supported at the moment)

Recommended configuration:

- Java 1.7 or 1.8
- 8 to 16 GB RAM
- 64-bit architecture
- AMD Radeon or NVIDIA GeForce family graphics card
- Microsoft Windows 7,8,10 (Linux or Mac OS X is only partially supported at the moment)

The Input Data Specifications

The COZOID is designed in such way that it accepts results produced directly by the existing docking tools, such as HADDOCK or PyDock. This means that the input data should consist of several files (e.g., in the PDB format), each describing single possible configuration (in this guide referred to as structure) computed by a docking tool and consisting of two molecular chains, each representing one of the interacting proteins.

Launching COZOID

After unzipping the cozoid.zip file, open the /bin folder and launch the corresponding executable file according to your version of the operating system.

User Interface

Main Menu

The main menu consists of the following categories:

File

- *Open Structure(s)…* - Allows the user to open one or more structures (i.e., configurations) from a file or folder stored on the hard drive.
- *Download Structure…* - Opens the dialog window enabling the user to enter a PDB code of a molecule, which will be downloaded from the PDB database.
- *Application Log* - Opens the panel with the information about the performed actions.
- *Application Settings* - Opens the general settings of the application.
- *Exit* - Closes the COZOID application.

View

- *Visualization Window* - Opens the central panel containing the 3D scene visualization.
- *Highlight Selection* - Enables/disables the highlighting of atoms, residues, or chains (according to the settings in the *Structure Selections* window) when hovering over with the cursor.
- *Surface Configuration* - Opens the *Surface Configuration* window in the bottom left panel. It allows the user to change the transparency of the molecular surface and the probe size, which controls the preciseness of the surface. It has no effect on other visualization styles.
- *Scene Fog* - Enables/disables the fog in scene, enhancing the depth perception.
- *Scene Drag Rotation* - When enabled, users can launch the automatic rotation of the whole scene. The direction and speed of the rotation are determined by dragging the mouse.
- *Orthographic Projection* - Allows the user to change the projection type to ortographics one.
- Reset Camera - Resets the scene so the loaded structures are centered to the viewport.
- *Reset Windows* - All windows are returned to their default position and size.

Visualization (this menu consists of two parts)

Protein Protein Interactions:

- *Residue Matrix* - Opens *Residue Matrix* window in the right panel.
- *Contact Zone Graphs* - Opens *Contact Zone Graphs* window in the right panel.
- *Protein Interactions 3D View Controls* - Opens *3D View Controls* window in the bottom left panel.

Structure Visualization Styles:

Allows users to change the visualization style of the activated protein structure(s).

- *Points* - Visualizes all atoms of the molecule as crosses positioned in the centers of these atoms.
- *Dots* - Displays the atoms as dotted spheres of van der Waals radii.
- *Wireframe* - Represents a molecule with its bonds visualized as lines.

- *Alpha Trace* - Visualizes the polypeptidic chain of the protein by connecting the C-alpha atoms.
- *Sticks* - Displays molecular bonds as three-dimensional sticks.
- *Balls & Sticks* - Shows both atoms and bonds of the protein.
- *Van der Waals Radii* - Molecules are represented by a set of spheres with van der Waals radii.
- *Cartoon* - Displays a representation of secondary structures.
- *Surface* - Displays a visualization of the protein surface. Its transparency can be set using the *Surface Configuration* window.

<u>Structure</u>

- *Overview* - Opens the top left panel containing the list of all loaded structures.
- *Selections* - Opens the top left panel containing the list of all created selections.
- *Search in structure…* - Opens the dialogue window enabling to search for residues and atoms in the selected structure.
- *Sequence* - Opens the bottom panel containing a sequential representation of all loaded proteins.
- *Properties* - Opens the top right panel, which contains the summarized information about the active structure.

<u>Help</u>

- *About COZOID* - Information about the product version and authors.

Top Toolbar

The top toolbar contains buttons with shortcuts to the most frequently used functions. See above for function descriptions.

<u>Structure opening:</u>

- *Open Structure(s)…*
- *Download Structure…*
- *Search in structure…*

<u>Structure Visualization Styles:</u>

- *Points*
- *Dots*
- *Wireframe*
- *Alpha Trace*
- *Sticks*
- *Balls & Sticks*
- *Van der Waals Radii*
- *Cartoon*
- *Surface*

<u>Other Functions:</u>

- *Colors* - Opens *Coloring* window, that enables users to change the structure coloring according to various properties.
- *Screen Capture* - Allows users to store the current snapshot of the visualization window.
- *Video Capture* - Allows users to store the video capture of the visualization window.
- *Fog* - Allows users to turn on and off the fog in the scene.

- *Selection Mode* - Allows users to create a new selection using rectangular, circular, or lasso selection mode in the Visualization window.

Main Windows

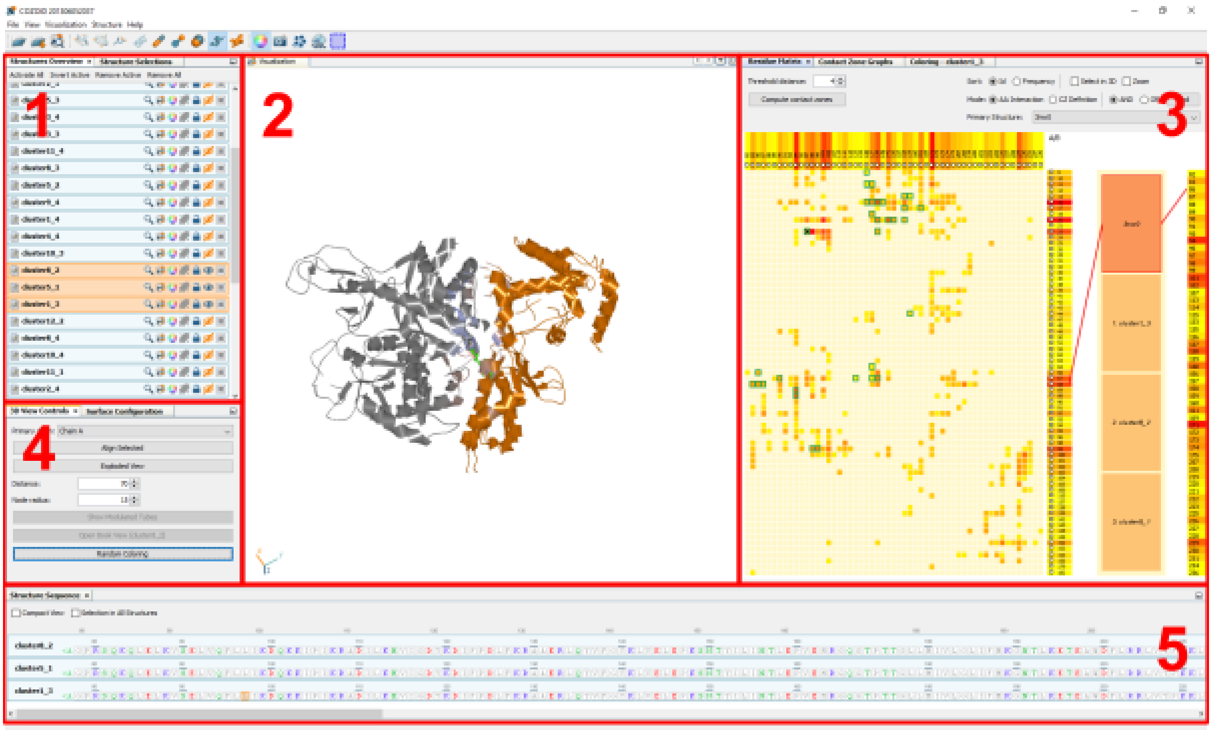

The default COZOID interface consists of the following parts:

1. Top left panel

   This panel contains two tabs.

   Structure Overview

   Structure Overview tab shows the list of all loaded files. Each file row providers users with the following functions:
   - Changing visualization style
   - Changing structure coloring
   - Locking structure in a given position so that the user cannot move the structure within the scene.
   - Show/Hide structure in the Visualization window
   - Closing structure (removes structure from the application)

   When clicking on a structure item with the right mouse button, a menu with additional functions appears.

   Structure Selections

   Structure Selections tab allows the user to create a selection of atoms, residues, and chains. This selection can be performed within one molecule or span more structures. The Selections window has the following functions:
   - *New…* - Allows the user to create a new selection with a user-specified name.
   - *Atoms* - Allows the user to select individual atoms.

- *Residues* - Allows the user to select whole residues.
- *Chains* - Allows the user to select the whole polypeptidic chains.

Each selection has its own record. The record contains:

- *Selection name* - Displays the name of the selection.
- *Changing visualization style* - Allows the user to change the visualization style of the selection. Supported styles are: Dots, Sticks, Balls & Sticks, Van der Waals Radii.
- *Selection color* - Allows the user to change the color of the selection.
- *Show/Hide selection* - Shows or hides the selection.
- *Lock selection* - Allows the user to lock the selection in a given position so the user cannot rotate and move the selection in the scene.
- *Close* - Removes the selection.

2. Visualization window

The visualization window displays all loaded structures (if they are not explicitly hidden) and allows the user to control them via the standard interface. The user can rotate (*left mouse button click* + *mouse drag*), scale (*right mouse button click* + *mouse drag*), or translate them (*mouse wheel button click* + *drag*).
The scene can be controlled using two different approaches:

- Global manipulation - users can manipulate (zoom, rotate, move) the whole scene (with all loaded structures at once). This can be done by using three mouse buttons.
  - *Left mouse button* - rotates all structures (active and inactive) around the scene center.
  - *Right mouse button* - zooms the whole scene.
  - *Middle mouse button* - translates the whole scene.
- Local manipulation - users can only manipulate active structures (highlighted in the Structures Overview window). This manipulation is activated by pressing CTRL + mouse buttons.
  - *CTRL + left mouse button* - rotation with the active structures around their local center (defined by the bounding box).
  - *CTRL + right mouse button* - zooms the active structures.
  - *CTRL + middle mouse button* - translates the active structures.

3. Right panel

This panel by default contains three tabs.

Residue Matrix

Residue Matrix tab enables the computation of the contact zones for loaded structures and subsequent exploration and filtering these contact zones.
This tab consists of two main parts:

- *Matrix* - The rows and columns of the Matrix view correspond to the interacting proteins (2 chains of the structure). The rows and columns are formed by residues from the interacting proteins which are in contact with at

least one loaded structure. The contact between the residues is based on their Euclidean distance. Two residues are considered to be in contact if their mutual distance is below a user defined threshold. The color of each cell in the matrix corresponds to the number of occurrences of the corresponding interacting residues in the set of loaded structures. The colored lists of residues at the axes of the matrix can be interpreted as histograms, encoding the number of their occurrences. The intense red color represents the pairs of residues that are interacting in most of the structures.

- *Side View* - The central part of the Side View shows a scrollable list of all visible structures sorted by the similarity with the primary structure (e.g., the crystal structure from the PDB database). The primary structure is always displayed as the first one on the top of the list. The Side View enables the iterative search through the list of structures and the exploration of all pairs of interacting residues for each structure. The user can select a structure in focus by clicking on it. By default, each structure in focus contains one polyline connecting those two residues from the contact zone which are the closest ones among all the possible pairs. The user can hover the mouse over the lists of residues on the left and the right side and inspect the corresponding connection lines for a given residue. By clicking on the rectangle representing a given residue, the connection lines remain in the view. The pairs of residues forming the structure in focus are highlighted in the matrix using green border rectangles.

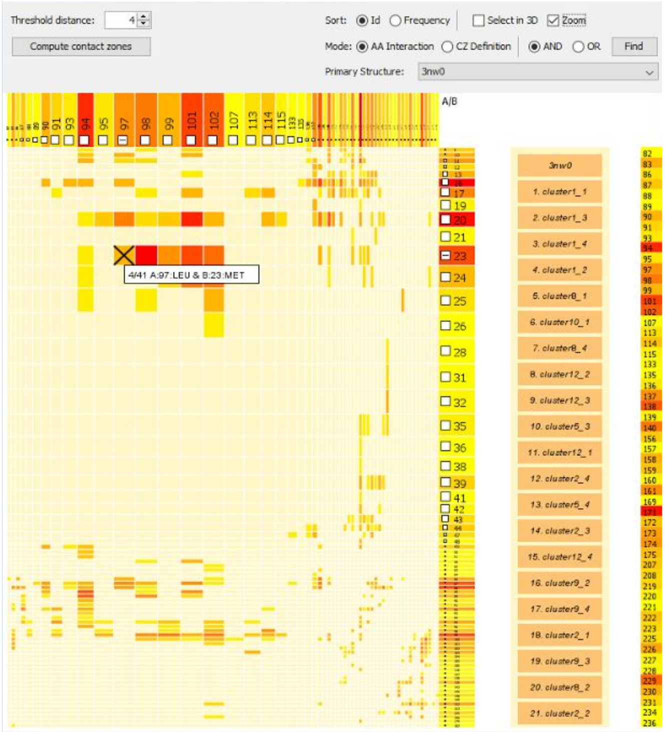

The following functions are provided for the exploration of the matrix view:

- *Threshold distance* - Sets the maximal distance between two residues to consider them as contact residues.
- *Compute contact zones* - Computes the contact zones of the loaded structures. Once the computation is finished, the matrix is displayed.

- *Sort* - Changes the order of residues in the Residue Matrix. It sorts the residues by their unique identifier. Frequency sorts the residues by the frequency with which their occur in the contact zones of the loaded structures.
- *Select in 3D* - Enables to select and highlight residues from the Residue Matrix in 3D (*CTRL + Right mouse button* on residue axes of Residue Matrix)
- *Zoom* - Enables lens-like zoom over the Residue Matrix if the matrix cells are too small.
- *Mode* - Sets filtering mode:
    - AA Interaction - Each selected cell of the matrix represents the interacting pair of residues (pair of residues in the interaction distance) that should be in the filtered structures.
        - *AND mode* - All selected interacting pairs should be present in the filtered structures.
        - *OR mode* - At least one of the selected interacting pairs should be present in the filtered structures.
    - CZ Definition - Enables to select individual residues on the axes of the matrix that should be present in the contact zones of the filtered structures without the constraint of precise interacting residue pairs.
        - *AND mode* - All selected residues should be present in the contact zone of the filtered structures.
        - *OR mode* - At least one of the selected residues should be present in the contact zone of the filtered structures.
- *Find* - Filters the loaded set of structures according to specified conditions.
- *Primary Structure* - Enables to select a primary structure to which the other structures are compared. The structures are then sorted according to similarity to the primary structure. This order is reflected in the matrix Side View.

Contact Zone Graphs

Contact Zone Graphs tab contains the Contact-Zone Lists of all active structures, which help to answer questions related to the comparison of the contact zones on the level of individual residues. The lists are computed automatically after the Matrix View is computed. It always shows the primary structure that can be selected in the drop down menu and all the active structures selected in the Structures Overview panel. The Contact-Zone List for one structure consists of two sets of residues in the contact zones, each set coming from one interacting protein. The left part of the list contains all residues coming by default from the reference protein (the primary chain of the structure), the right part is formed by their interaction counterparts in the paired protein (the secondary chain of the structure). The left part of the list can be sorted according to the physico-chemical properties of the depicted residues. The color of the rectangles depicting the residues also reflects these properties. White rectangles denote residues that are present in the contact zone of the primary structure but are missing in the contact zone of the structure depicted by the given list. The red border and connection lines between rectangles denote the residues and interaction bonds that are present in both the primary structure and structure depicted in the list. By

clicking on individual rectangles, the corresponding residues are selected in the 3D view as well.

The following functions are provided for the exploration of the lists:

- *Primary structure* - Allows selecting the primary structure. It is always shown on the right side.
- *Chain A, Chain B* - Allows the user to select primary chain, i.e., the structure chain which will be aligned if the alignment is activated.
- *Sort by* - Allows changing the vertical ordering of residues in the view based on a selected property.
- *Shrinked view* - In this mode, the selected view is minimized as much as possible: the empty space between the residues is removed and also only present residues are shown (i.e., the missing residues are not displayed)
- *All contacts* - All selected residues in contact are shown; otherwise only the closest contacts are shown.
- *Min. Size* - Sets the minimum size of the rectangles representing the residues.

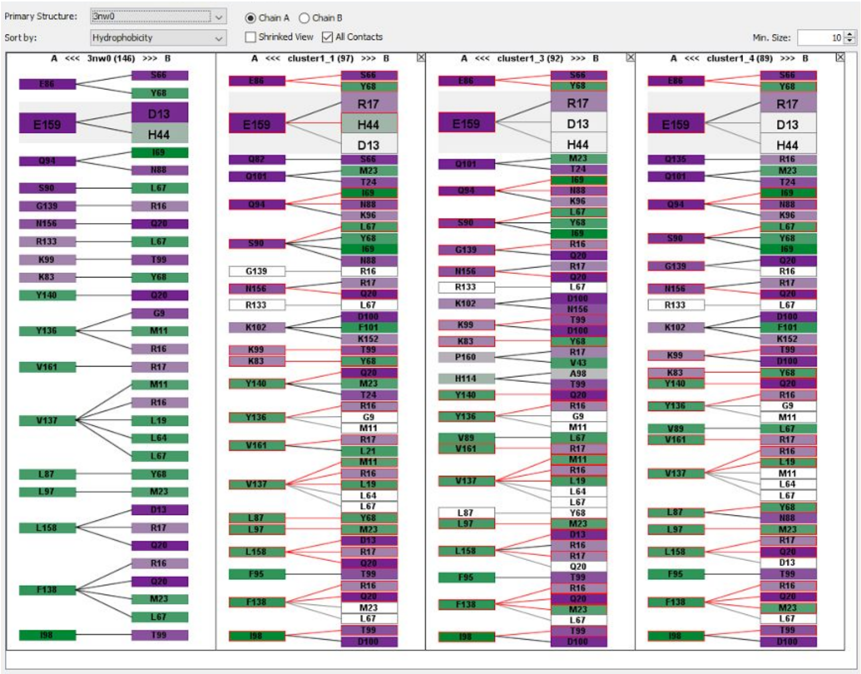

Coloring

The Coloring tab is used to color the loaded structures and their individual parts (Atoms, Residues, Chains, Secondary Structures, Contact zones, or whole structures). For each part there are several color schemes available, for example, hydrophobicity of residues. Global setting enables to change the background of the whole scene.

4. Bottom left panel

This panel contains two tabs.

3D View Controls

The 3D View Controls tab enables the activation of functions related to 3D visualizations of the Contact Zones. The Residue Matrix must be computed to activate these views.
It consists of the following parts.

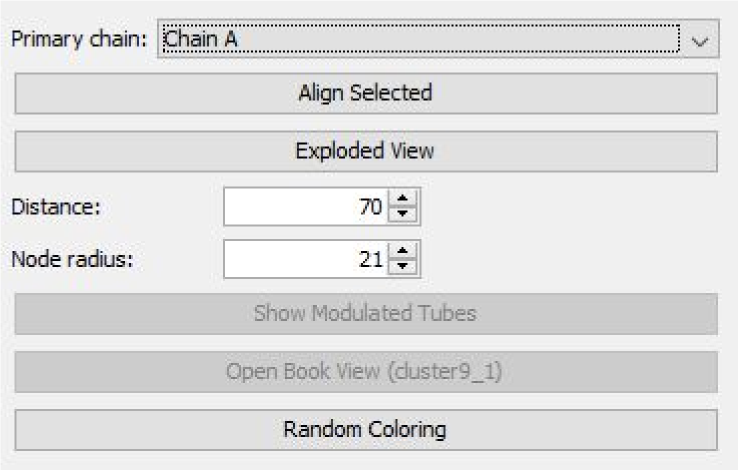

- *Primary chain* - Allows the user to select the primary chain, i.e., the structure chain which will be aligned if the alignment is activated.
- *Align Selected* - Aligns all active structures based on the selected primary chain.
- *Exploded View* - Activates/Deactivates the Exploded View of the active structures. It enlarges the distance between the interacting proteins so that the paired proteins (secondary chains) are placed onto a regular grid and do not overlap.
- *Distance* - Allows the user to control the distance between the reference and paired proteins in the Exploded View.
- *Node radius* - Allows the user to control the distance between individual paired proteins in the Exploded View.
- *Show Modulated Tubes* - Shows/Hides the connection between the corresponding contact zones on the reference and paired protein in the Exploded View.
- *Open Book View* - Activates/Deactivates the Open Book View. Rotates the contact zone of the reference and paired protein of the selected structure towards the camera, so both parts of the contact zone are visible. Only possible when only one structure is active.
- *Random coloring* - Assigns random colors to individual contact zones of all loaded structures. The parts of the structures that are not in the contact zones are colored uniformly by the chain.

Surface Configuration

The Surface Configuration tab directly influences the computed and visualized surface of the selected structure. It allows to change the transparency of the surface for a selected structure and change the probe size for a structure's surface. This way, the overlapping Contact Zones can be compared.

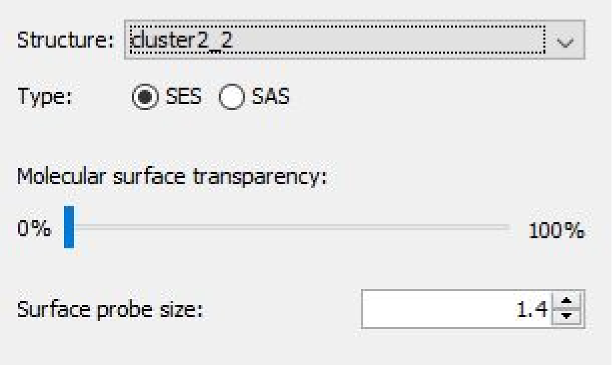

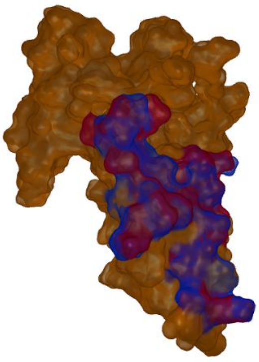

5. Bottom panel

Structure Sequence

Bottom panel contains the *Structure Sequence* window. This window shows the primary structure of all loaded structures. Each row consists of the following sections:

- Structure name
- List of one-letter abbreviations of residues

Clicking the right button mouse on a residue shows the list of all atoms of a given residue.

Guided Example

Tha exemplary data (http://decibel.fi.muni.cz/cozoid/data.zip) can be loaded via the Open Structure(s)... dialogue located in the File menu, where multiple files in traditional molecular representation file formats can be selected. Here we select a set of 40 docking results of NSE1 - NSE3 proteins computed by the HADDOCk tool and the crystal structure 3NW0 of the docked proteins.

IMPORTANT NOTE: In the provided test dataset we use the edited version of the 3NW0 structure, which contains flipped chains. This is due to the fact that the HADDOCK tool used to generate the docking configurations flipped the chains of the 3NW0 during the docking process.

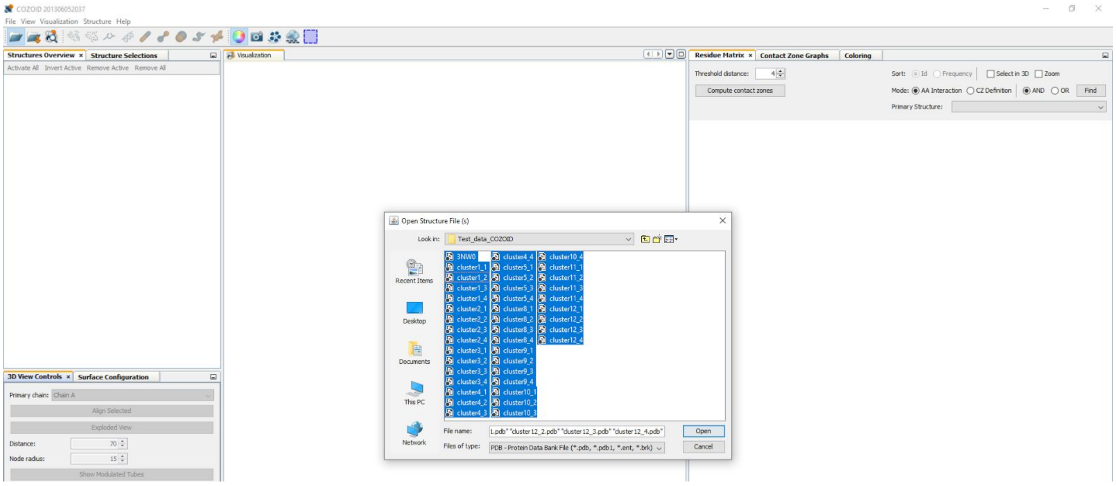

The loaded files are displayed in Structure Overview panel on the left side of the application window. The 3D representation of the loaded files is displayed in the Visualization window. Upon addition of new file, the Residue Matrix panel is activated.

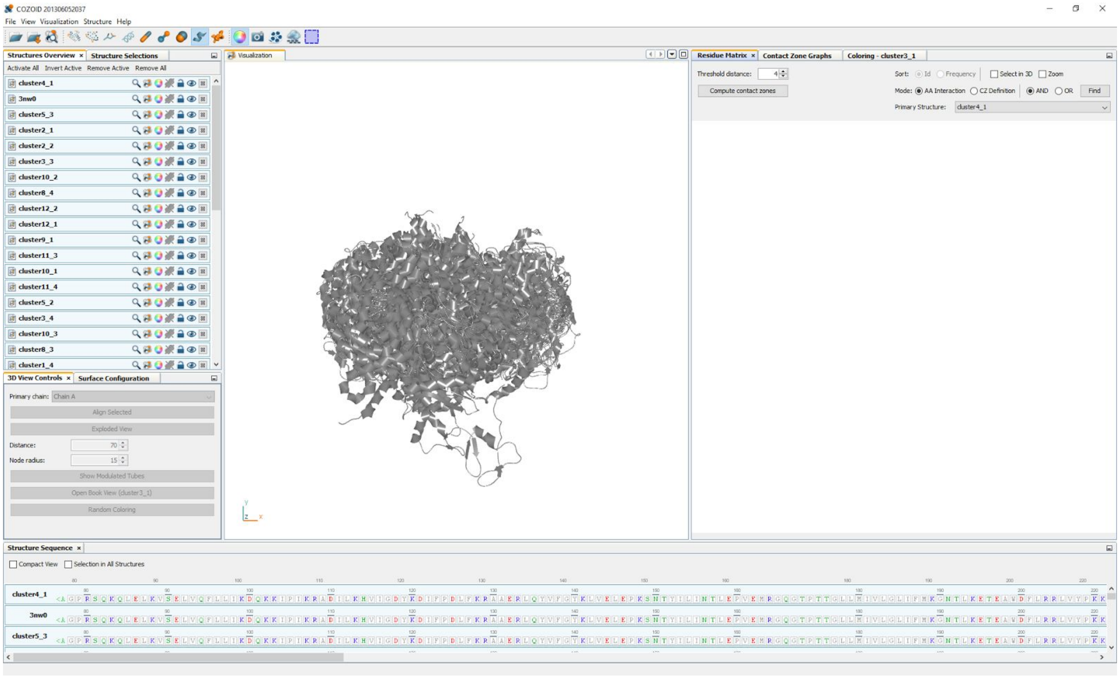

We click on “Compute contact zones” to compute the Residue Matrix. Once the Matrix is computed, we select the 3NW0 structure as the primary structure to see which docking results are the most similar ones to this structure.

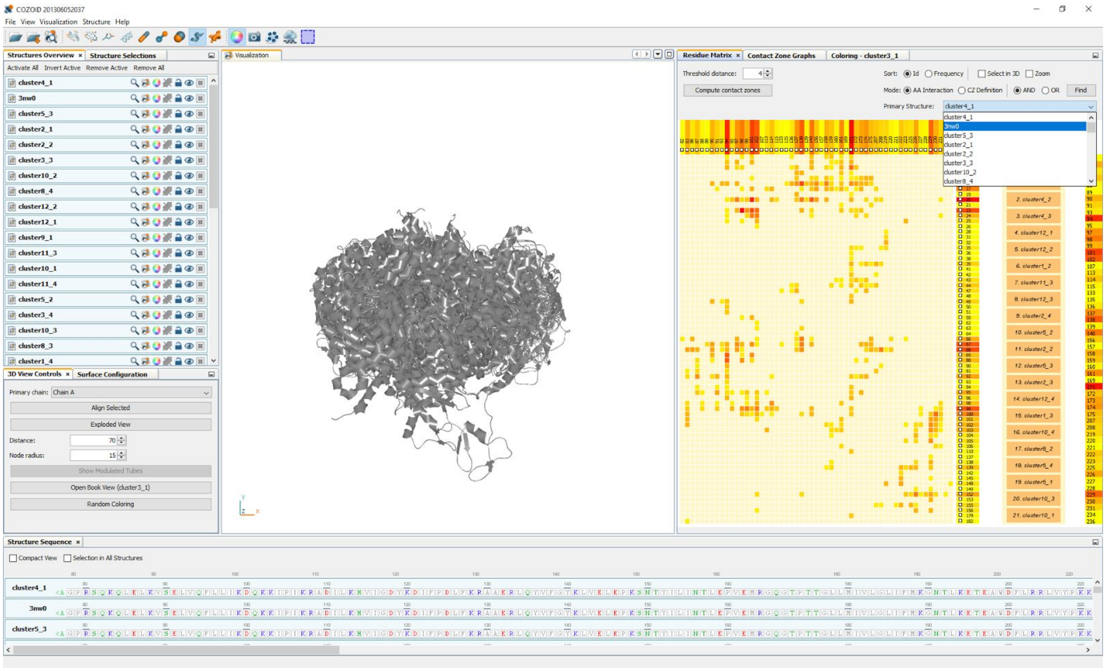

Next we can utilize the Matrix Side View to browse the most similar structures. We select a structure in the side view by clicking on it. We can then see the closest residue pair in the contact zone of this structure. By hovering over the list of residues lining the Side View, we see the interacting pairs that are present in this structure. These are also highlighted by green rectangles in the Matrix View.

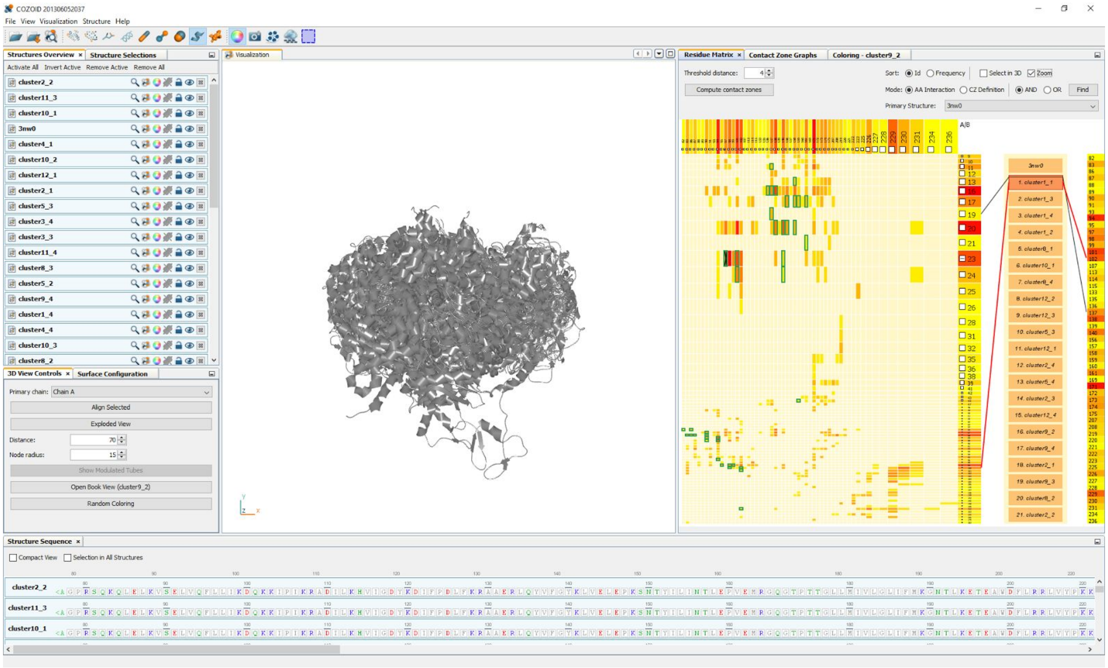

We now want to utilize the experimentally gained knowledge about the contact zone between the two proteins. We know that the pair of residues leucine 97 and methionine 23 should interact. Therefore, we select the cell in the Residue Matrix representing this interaction pair. We can see, that this pair is present in four loaded structures.

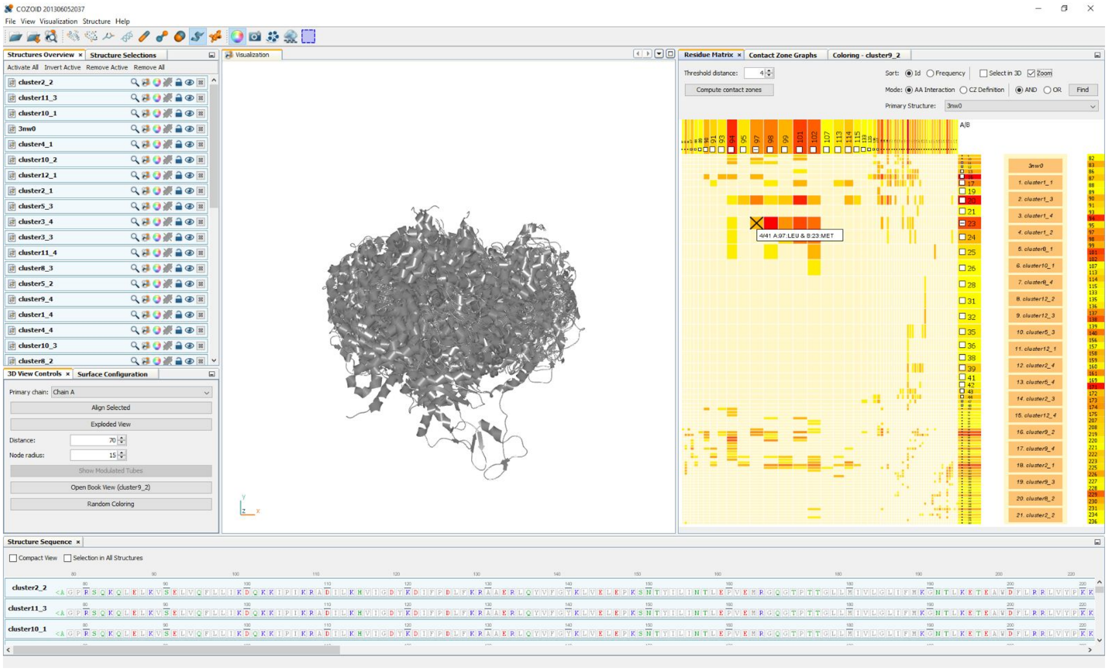

We want to reduce our set of structures to these four structures. Upon clicking on “Find”, the structures that do not contain this interaction pair of residues are hidden and the remaining four structures are activated. We can now see that the four structures include the crystal structure and three structures that were evaluated as the most similar ones. In the 3D view, the defined interaction pair was selected and highlighted for each structure.

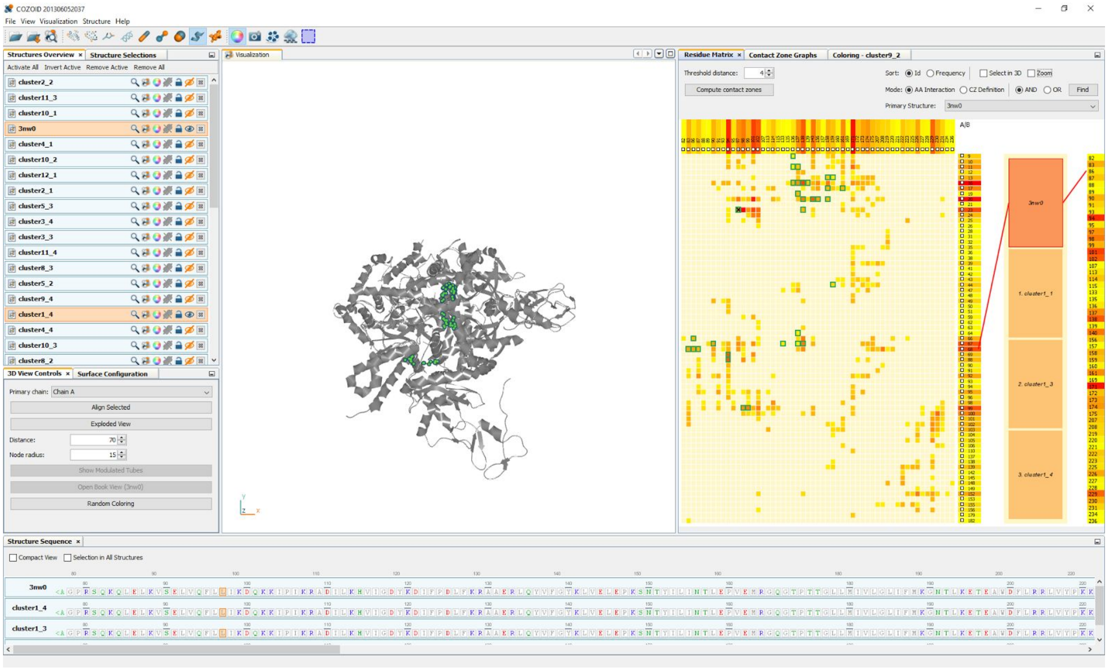

We now want to explore the selected structures in the 3D view. We can see that the structures are misaligned. Therefore, we select the “Align” option in the 3D View Controls. This aligns the loaded structures to one of the chains (the primary chain selected in 3D View Controls).

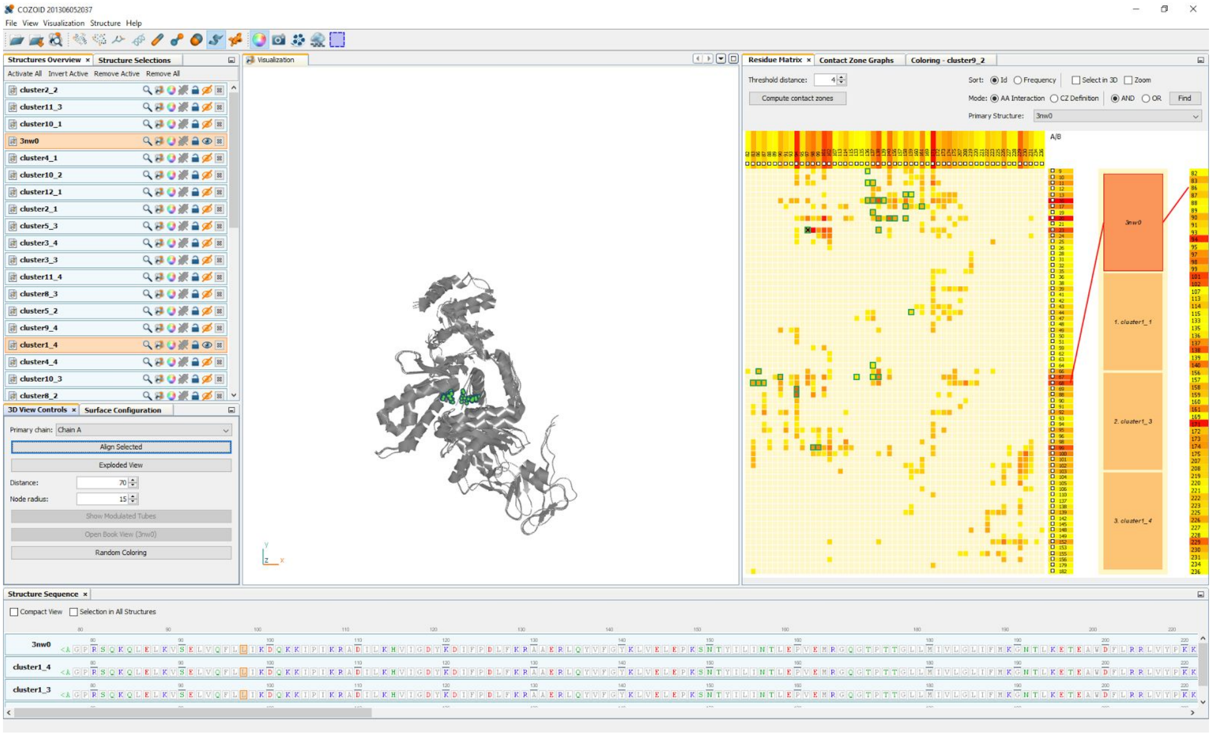

The structures are now aligned. However, it is still difficult to identify individual protein chains as well as the contact zones between them. Therefore, we choose the option “Random Coloring”, which assigns a random color to the contact zones of individual structures.

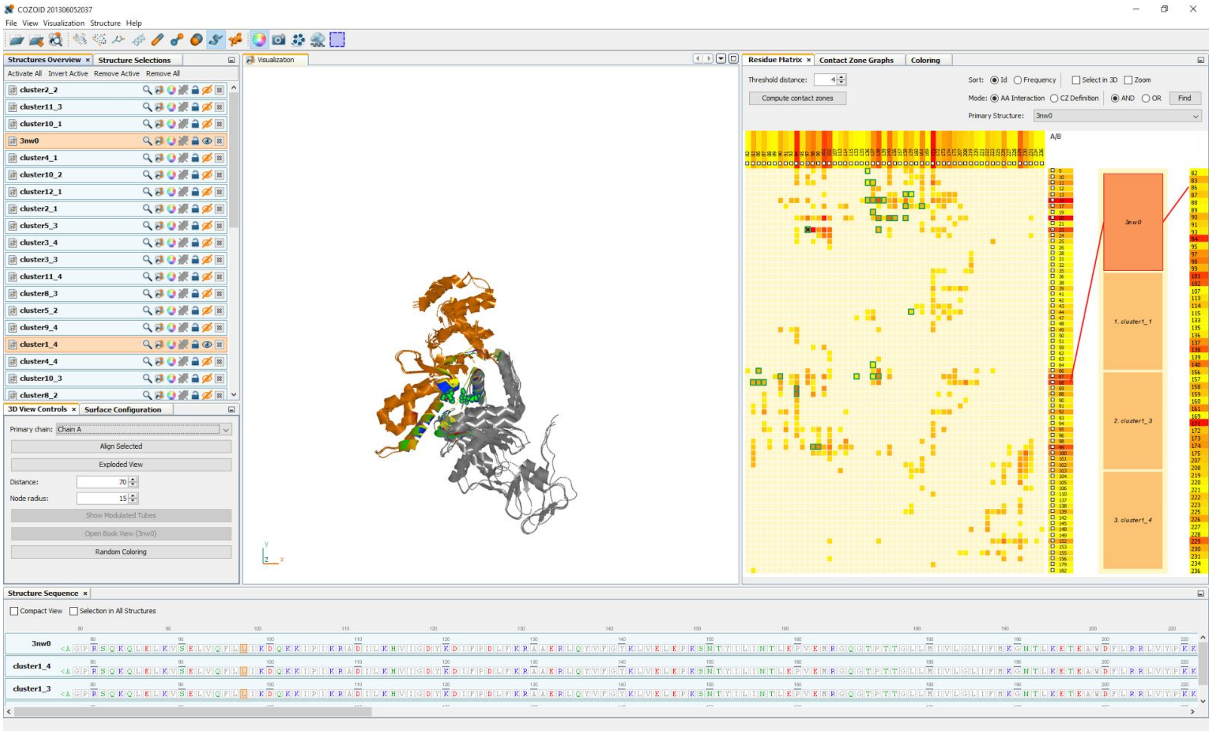

We can also adjust the structure visualization type to a more suitable representation, e.g., the surface representation.

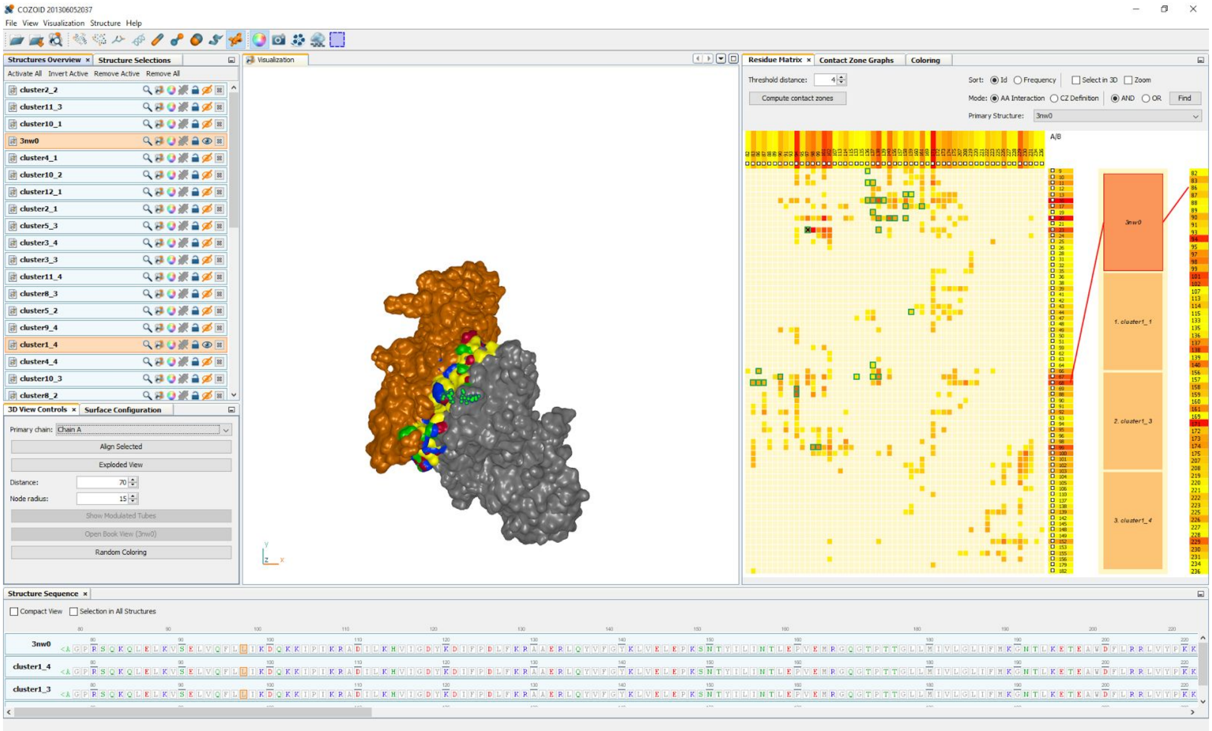

However, the contact zones between the interacting proteins are still occluded and difficult to explore. Therefore, we select the “Exploded View”, which enlarges the distance between the aligned reference proteins and paired proteins.

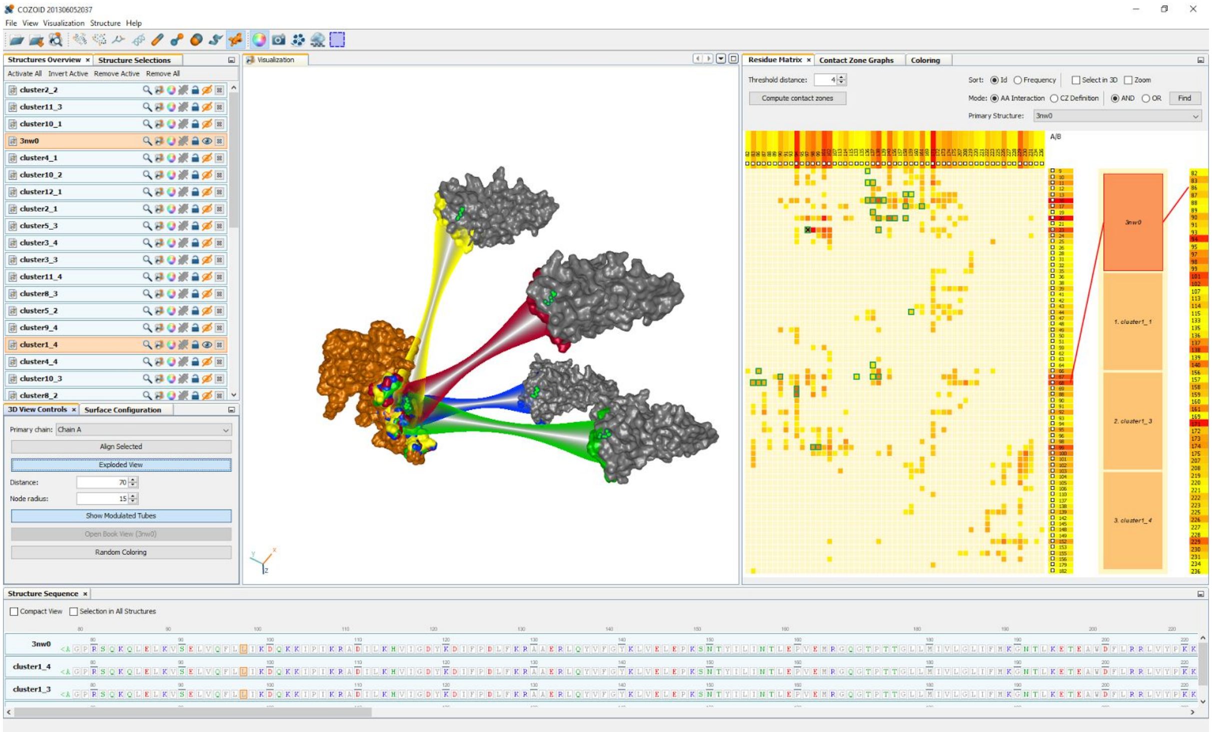

The proteins are initially connected by the colored tubes connecting the corresponding contact zones. However, it is possible to switch them off to get a better view of the overlapping contact zones in the reference proteins.

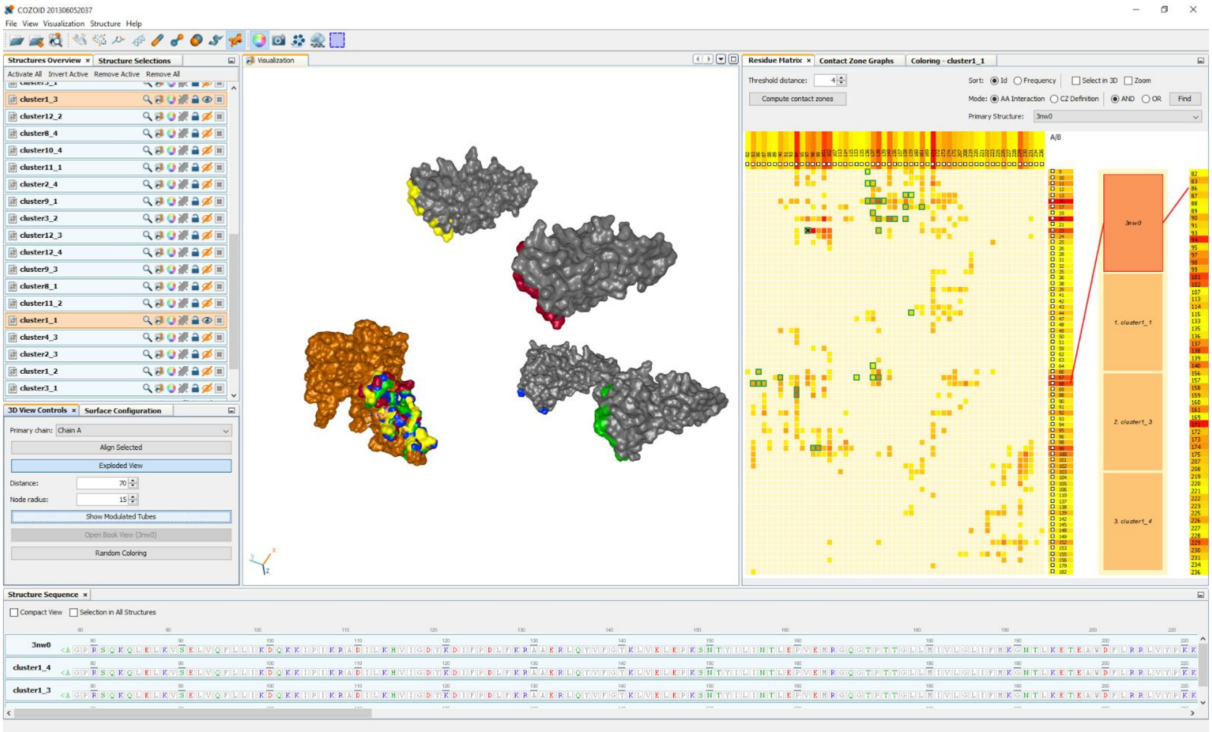

Also the transparency settings in the Surface Configuration can be utilized to change the opacity of structure surfaces and to better see the overlapping contact zones.

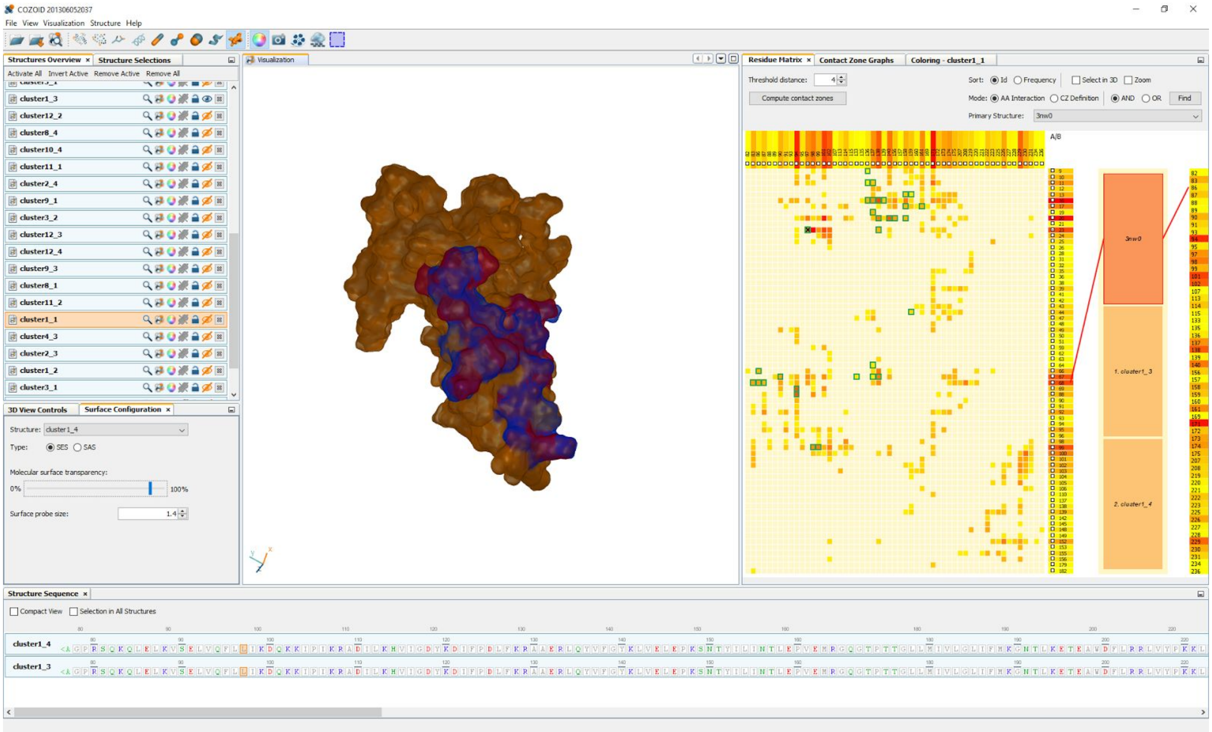

If we want to explore only one structure contact zone in detail, we can deactivate all other structures and select the “Open-Book View”. This rotates the contact zones of one structure towards the camera.

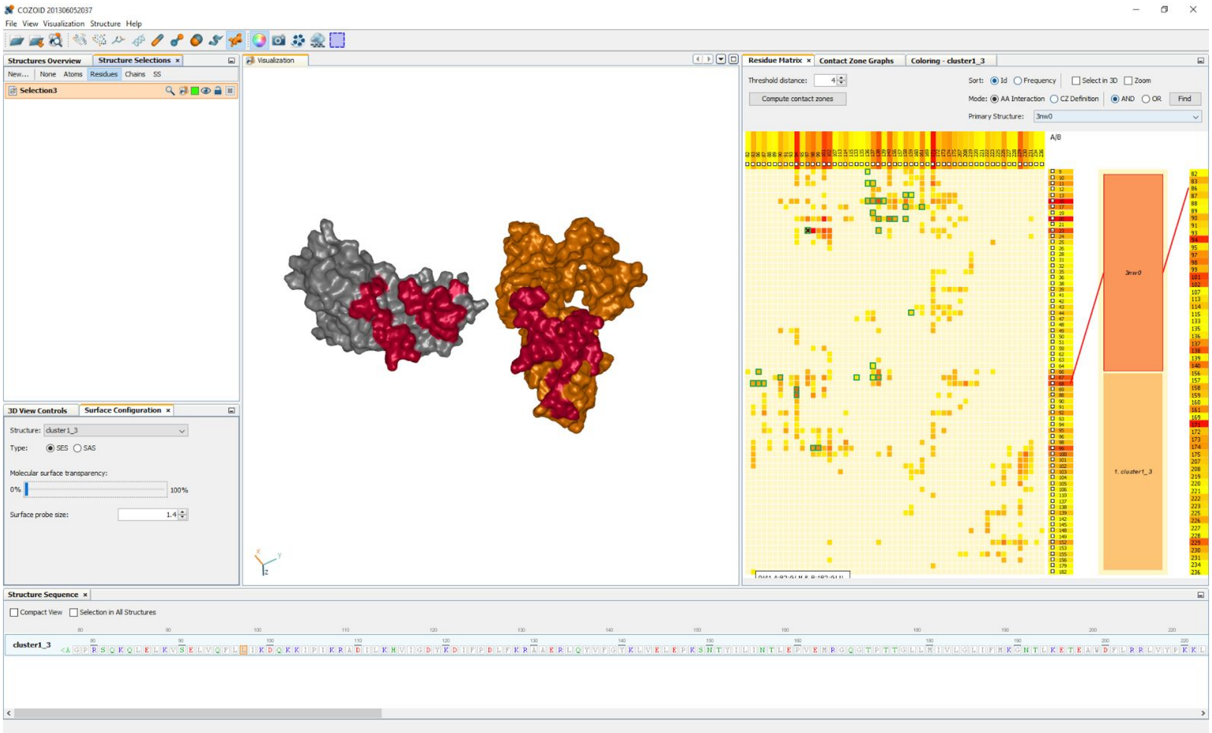

Subsequently, the contact zone can be colored by various properties, such as the distance between the residues or their hydrophobicity, using the Coloring window.

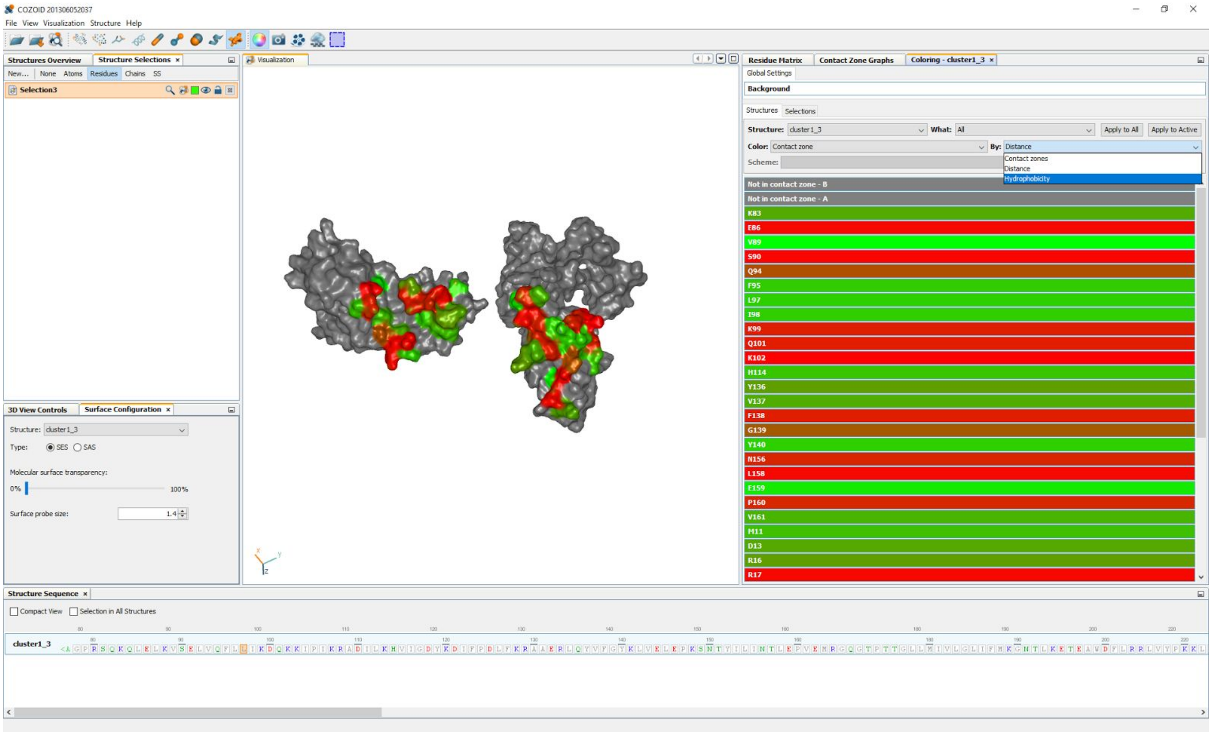

If we want to compare the contact zone on the level of individual residues, we can switch to the Contact Zones Graphs where we see the similarities and differences of the contact zones in detail. The Contact-Zone Lists are again sorted by similarity.

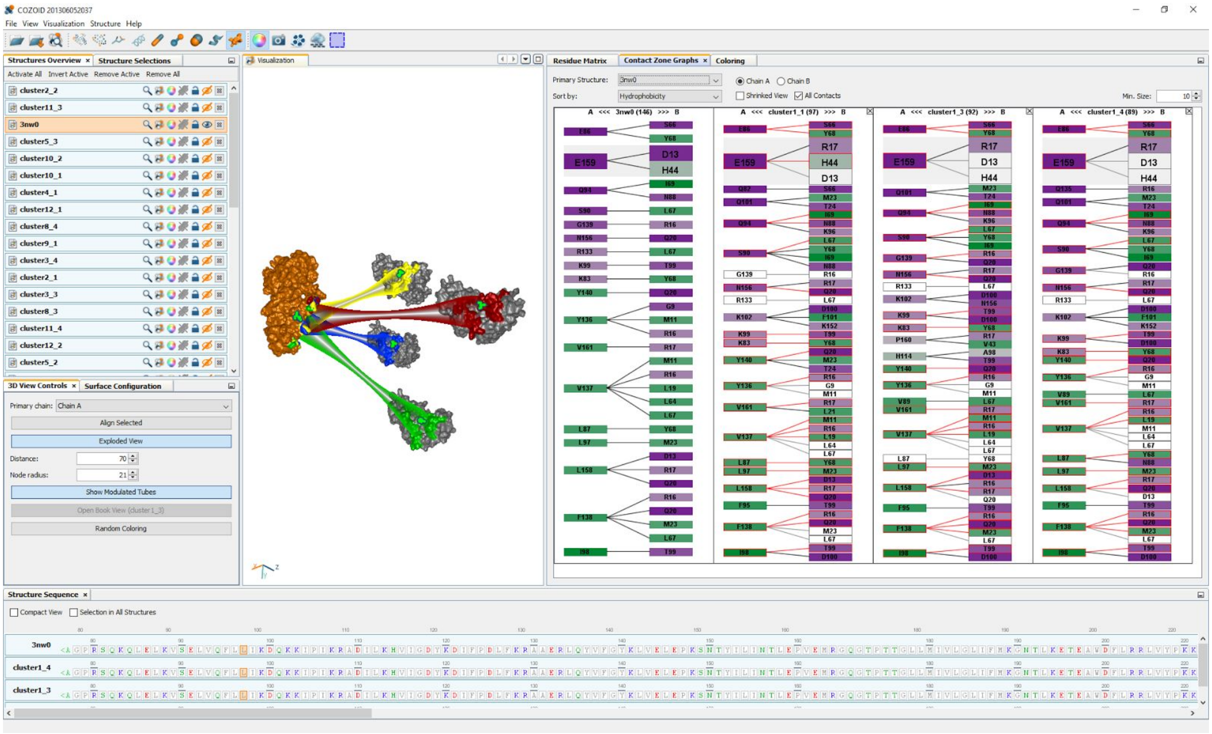

Supplement: Supplementary file 4 — User guide. User guide for the software tool. (PDF 3502 kb) [file 12859_2018_2113_MOESM4_ESM.pdf]
